# C-reactive protein in cardiovascular disease: clinical associations, conformation-dependent biology, and therapeutic perspectives

**DOI:** 10.3389/fimmu.2026.1924339

**Published:** 2026-08-25

**Authors:** Bingxing Gong, Haiqin Xu, Honglan Liu, Mengmeng Wang, Kailin Yang, Junpeng Chen, Xiaoyang Guo, Tianqing Zhang, Liangqing Ge

**Affiliations:** 1 Department of Cardiology, Changde Hospital, Xiangya School of Medicine, Central South University (The First People’s Hospital of Changde City), Changde, China; 2 Department of Cardiac Electrophysiology, Changde Hospital, Xiangya School of Medicine, Central South University (The First People’s Hospital of Changde City), Changde, China; 3 Institute of Basic Research in Traditional Chinese Medicine, Hunan Academy of Chinese Medicine, Changsha, Hunan, China; 4 Center for Cardiometabolic Science, University of Louisville, Louisville, KY, United States; 5 Department of Mathematics, College of Science, City University of Hong Kong, Hong Kong, Hong Kong SAR, China

**Keywords:** cardiovascular disease, C-reactive protein, CRP conformation, inflammation, residual inflammatory risk, risk stratification

## Abstract

C-reactive protein (CRP) is a widely used biomarker of systemic inflammation and cardiovascular risk. Its clinical and experimental relevance has been investigated across major cardiovascular disease (CVD) phenotypes, including coronary artery disease, heart failure, arrhythmias, cardiomyopathies, hypertension, and aortic aneurysm. High-sensitivity CRP (hs-CRP) assays detect low-grade systemic inflammation, and elevated circulating hs-CRP is associated with incident cardiovascular events, recurrent events, and mortality. CRP also exhibits conformation-dependent biological properties. Circulating pentameric CRP (pCRP) can undergo transition through modified pentameric CRP (pCRP*) to monomeric CRP (mCRP) at activated or damaged membranes. Experimental studies indicate that pCRP* and mCRP may exert localized effects on endothelial cells, vascular smooth muscle cells, immune cells, complement activation, coagulation, and extracellular matrix remodeling. However, observational, genetic, animal, and interventional evidence does not establish circulating CRP as a major independent cause of CVD, and the causal and clinical significance of tissue-associated CRP conformations remains unresolved. Clinically, hs-CRP- and CRP-based composite indices may support risk stratification and assessment of residual inflammatory risk, although their performance and thresholds vary across populations and clinical settings. Upstream anti-inflammatory and metabolic therapies can lower circulating CRP indirectly, whereas CRP apheresis, anti-CRP antibodies, and inhibitors of conformational transition are direct CRP-directed approaches that remain investigational. This review synthesizes clinical, structural, mechanistic, and therapeutic evidence for CRP across major CVD phenotypes and identifies priorities for conformation-specific measurement, causal evaluation, and target-specific intervention. At present, hs-CRP is principally a biomarker of inflammatory risk, whereas the therapeutic value of directly targeting CRP remains to be established.

## 1 Introduction

Cardiovascular disease (CVD) remains the leading global cause of mortality and disability. Against the backdrop of population aging and the rising prevalence of metabolic disorders, the burden of CVD continues to escalate, severely threatening human health and imposing substantial strain on healthcare systems worldwide (1). Atherosclerosis constitutes a major pathological basis for coronary heart disease, ischemic stroke, and peripheral artery disease and contributes substantially to the development of ischemic heart failure. Elucidating its mechanisms and advancing targeted interventions have therefore remained central priorities in cardiovascular research. Although traditional risk factors such as dyslipidemia, hypertension, hyperglycemia, and smoking account for a considerable proportion of CVD incidence, they cannot fully explain the progression of subclinical lesions, residual cardiovascular risk, or variations in susceptibility across populations. This gap points to additional biological pathways that require deeper investigation (2). In recent years, extensive basic and clinical studies have identified chronic low-grade systemic inflammation as an important contributor across the continuum of atherosclerosis—from endothelial injury and lipid deposition to plaque growth, inflammatory amplification, and eventual rupture with thrombosis. It provides an important mechanistic link between conventional risk factors and subsequent cardiovascular events (3, 4).

C-reactive protein (CRP), a classic acute-phase reactant synthesized predominantly in the liver in response to cytokines such as interleukin-6 (IL-6), is among the most widely used systemic inflammatory biomarkers in clinical practice (5). The development of high-sensitivity CRP (hs-CRP) assays has enabled reproducible quantification of the low circulating CRP concentrations associated with subclinical, low-grade inflammation (6). Through decades of translational validation, CRP—particularly when measured using hs-CRP assays—has become an established biomarker for cardiovascular risk stratification and prognostic assessment. Experimental studies also suggest that conformationally altered CRP may exert biological effects within diseased tissues, although the clinical relevance and causal contribution of these effects remain uncertain (7). Large observational studies have consistently associated elevated circulating hs-CRP with incident cardiovascular events and mortality, often independently of conventional risk factors. In contrast, Mendelian randomization studies have generally not demonstrated a consistent causal effect of genetically predicted circulating CRP on most major CVD outcomes. This discrepancy underscores the need to distinguish the prognostic value of hs-CRP from evidence that CRP itself directly causes cardiovascular injury (8, 9).

At the molecular level, CRP exists in three functionally relevant conformational forms—native pentameric CRP (pCRP), a conformationally modified pentameric intermediate (pCRP*), and monomeric CRP (mCRP)—with distinct distributions and biological activities (10). Circulating CRP exists predominantly as relatively stable pCRP. At sites of cellular or tissue injury, membrane binding and local microenvironmental conditions can induce conformational rearrangement of pCRP to pCRP*, which exposes previously concealed proinflammatory binding sites while retaining the pentameric scaffold and may precede further dissociation into mCRP (11). The pentameric form primarily supports basal immune regulation and ligand recognition (12). In contrast, experimental studies indicate that tissue-associated mCRP can promote endothelial dysfunction, stimulate vascular smooth muscle cell activation, enhance lipid accumulation within the vessel wall, activate the complement cascade, and amplify local inflammatory responses. These observations provide mechanistic plausibility for a local role of conformationally altered CRP in vascular injury but should not be extrapolated directly to the biological activity of circulating CRP measured by hs-CRP assays (13). Importantly, routine CRP and hs-CRP assays do not provide conformation-specific quantification and therefore cannot distinguish circulating pCRP from pCRP* or mCRP.

Beyond single-marker strategies, investigators have constructed composite indices that integrate inflammatory and metabolic signals, including the hs-CRP to high-density lipoprotein cholesterol (HDL-C) ratio and the CRP-triglyceride-glucose index. By simultaneously capturing inflammatory activation and metabolic disturbance, these indices may improve the identification of subclinical atherosclerosis and residual cardiovascular risk compared with the use of CRP or lipid parameters alone. However, their incremental clinical value, optimal thresholds, and generalizability require further validation (14, 15).

In clinical translation and targeted therapy, landmark trials such as the Canakinumab Anti-inflammatory Thrombosis Outcomes Study (CANTOS) and cardiovascular outcome trials of colchicine have demonstrated that inhibition of selected upstream inflammatory pathways can reduce cardiovascular events in appropriately selected patients, establishing inflammation as a modifiable component of residual cardiovascular risk (3, 16). These interventions do not directly target CRP; rather, reductions in circulating CRP occur downstream of their effects on inflammatory signaling. Additional work has linked the cardiovascular benefits of established therapies, including statins and glucagon-like peptide-1 receptor agonists (GLP-1RAs), to their capacity to lower CRP levels and interrupt inflammatory signaling. Reductions in CRP may therefore provide a useful measure of treatment-associated changes in inflammatory burden, although they do not establish that CRP lowering mediates the clinical benefits of these therapies (17). For prognosis, persistently elevated hs-CRP concentrations are associated with disease progression, recurrent cardiovascular events, and mortality. CRP assessment may therefore complement established approaches to risk stratification in asymptomatic high-risk individuals and prognostic evaluation in patients with confirmed CVD, providing additional information across the spectrum of disease management (18, 19).

Despite substantial progress in research on the relationship between CRP and CVD, several fundamental controversies and unresolved scientific questions continue to limit precise clinical translation. First, the causal mechanisms underlying the association between CRP and CVD onset remain incompletely elucidated. Although observational and experimental studies support potential biological involvement, genetic evidence has generally challenged a direct causal role for circulating CRP. Whether locally deposited, conformationally altered CRP contributes to disease independently of systemic inflammation remains unresolved (20, 21). Second, the predictive performance of CRP exhibits marked heterogeneity across populations. Risk thresholds and prognostic value vary considerably by race, age, sex, and comorbid conditions, and universally applicable stratification standards are still lacking (22, 23). Third, the optimal timing, effective dosing windows, and long-term safety of inflammation-targeted interventions selected or monitored using hs-CRP remain unclear. Approaches that directly remove or inhibit CRP, including CRP apheresis and conformation-specific inhibitors, remain investigational and should be distinguished from therapies that indirectly reduce CRP by acting on upstream pathways (24). In addition, genetic polymorphisms, systemic immune status, and metabolic disorders can substantially influence CRP concentrations and inflammatory responses, thereby increasing the complexity of individualized therapeutic strategies (25). Fourth, the clinical utility, diagnostic cutoffs, and long-term prognostic value of novel composite inflammatory-lipid markers have not yet been validated in large multicenter studies with extended follow-up, and these markers have not been incorporated into routine clinical practice (26).

Building on the current research landscape and these identified bottlenecks, this review summarizes advances over the past decade in CRP structural biology, conformation-dependent biological effects, and clinical associations across major CVD phenotypes. Emphasis is placed on distinguishing circulating hs-CRP measurements from the experimental and tissue-level evidence concerning pCRP, pCRP*, and mCRP, as well as on the interaction between inflammation and metabolic dysfunction. The review further assesses the utility of CRP and emerging composite markers across the clinical continuum, from early screening and risk stratification to inflammation-targeted treatment and prognostic evaluation. Concurrently, it addresses existing academic controversies, research gaps, and translational challenges while outlining promising future directions, including conformation-specific therapeutic targeting and personalized inflammation-based approaches. The aim is to provide an evidence-based and conformation-aware framework for interpreting the roles of CRP as a clinical biomarker and potential local biological effector, thereby supporting the refinement of more precise, inflammation-oriented strategies in CVD.

## 2 Structural characteristics and conformation-dependent biological properties of CRP

CRP is a classical calcium-dependent short pentraxin that circulates predominantly as a pentamer and is an established biomarker of the acute-phase response. Since its discovery in 1930, scientific understanding of its biology has evolved considerably. Initially viewed primarily as an inflammatory marker, CRP is now recognized not only for its contributions to immune regulation but also for its potential roles in vascular homeostasis and disease pathology, positioning it as a key focus in cardiovascular inflammation research (27, 28). Relative to traditional inflammatory indicators, CRP possesses distinct biological advantages, including a stable plasma half-life, sensitive responsiveness to inflammation, and high reproducibility of detection. Advances in high-sensitivity assays have enabled reproducible quantification of subclinical low-grade inflammatory states, thereby providing an important foundation for cardiovascular risk assessment and prognostic evaluation in CVD (6, 29). In recent years, progress in structural biology, molecular genetics, and translational research has substantially clarified the differential functions of CRP conformational forms, their molecular regulatory networks, and interaction mechanisms. These developments have challenged the earlier perspective of CRP as merely a non-specific inflammatory marker and have prompted renewed investigation into whether conformationally altered CRP acts locally within diseased cardiovascular tissues (24, 30).

### 2.1 Molecular structure and conformational characteristics of CRP

**Figure 1:**
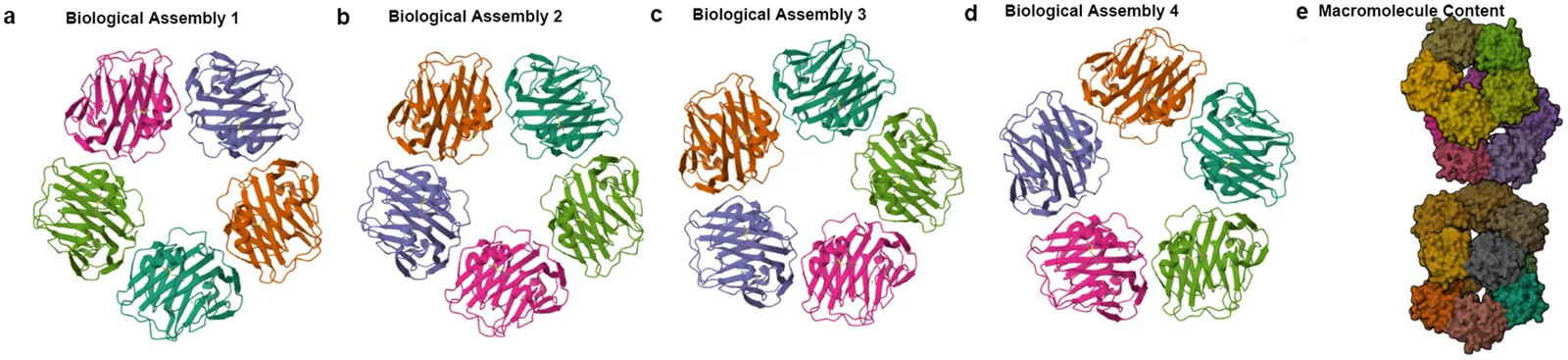
Structural organization and crystallographic assemblies of human C-reactive protein. Panels **(a–d)** depict the four author-assigned biological assemblies in the zinc-containing human CRP crystal structure deposited as PDB entry 3PVN. Each assembly is a C5-symmetric homopentamer composed of five identical CRP subunits. Panel **(e)** shows the complete macromolecular content of the deposited structure, comprising 20 CRP subunits with a total modeled mass of approximately 463.29 kDa. Structural images were adapted from the RCSB Protein Data Bank (RCSB.org), PDB ID 3PVN, using the Mol* molecular graphics viewer (32). The corresponding crystallographic study was reported by Guillon et al. (33). RCSB.org screenshot content is used with attribution under CC BY 4.0; accessed August 5, 2026.

Mature human CRP is a non-glycosylated globular protein whose core structure consists of five identical polypeptide subunits assembled non-covalently into a cyclic pentameric ring. The molecule adopts a disk-like symmetric configuration with a characteristic central pore and has a total molecular weight of approximately 115 kDa. Each subunit contains 206 amino acid residues with a molecular weight of approximately 23 kDa (5, 31). Every subunit possesses two functionally distinct structural interfaces and calcium-binding sites. This highly conserved architecture with clear functional partitioning provides the structural foundation for CRP’s multiple biological activities (12). **Figure 1** presents the crystallographic organization of human CRP in the zinc-containing structure deposited as Protein Data Bank (PDB) entry 3PVN. Panels a-d depict four author-assigned C5-symmetric homopentameric assemblies, whereas panel e shows the complete macromolecular content of the deposited structure, comprising 20 CRP subunits (33).

One face of each subunit contains a double calcium-ion binding pocket. Calcium coordination stabilizes the protein’s spatial conformation and enables specific binding to phosphatidylcholine on cell membranes, pathogen-derived phospholipids, and debris from damaged cells, thereby mediating innate immune recognition (12). The opposite effector face contains sites implicated in interactions with complement component 1q (C1q) and Fc receptors. These spatially separated recognition and effector surfaces enable ligand-bound CRP to engage downstream immune pathways (12).

Current understanding recognizes three functionally distinct conformational forms of CRP: native pentameric CRP (pCRP), the conformationally modified pentameric intermediate pCRP*, and monomeric CRP (mCRP). These forms are linked through a sequential conformational transition in which membrane-bound pCRP first undergoes structural rearrangement to pCRP*, while retaining overall pentameric symmetry, and may subsequently dissociate into mCRP. The exposure of previously concealed neoepitopes during this transition contributes to their different ligand-binding and inflammatory properties (11).

Under physiological conditions, circulating CRP exists predominantly as native pCRP. This form is structurally stable with limited intrinsic proinflammatory activity in the absence of ligand binding. Experimental studies indicate that pCRP can bind circulating toxic histones and inhibit amyloid fibril formation, potentially limiting their cytotoxic effects (34, 35). At sites of tissue injury, pCRP can bind phosphocholine-containing membranes on activated platelets, damaged cells, or cell-derived microvesicles. Membrane binding and local physicochemical conditions promote the initial formation of pCRP* and can facilitate subsequent dissociation into mCRP (11, 36).

Experimental evidence indicates that tissue-associated pCRP* and mCRP exhibit greater proinflammatory activity than soluble circulating pCRP. Following conformational rearrangement, their ligand-binding capacity and cell-activating effects are markedly enhanced. mCRP can specifically interact with receptors on vascular endothelial cells, smooth muscle cells, and macrophages, thereby activating proinflammatory, pro-oxidative, pro-apoptotic, and pro-proliferative signaling pathways (24, 37). The transitional pCRP* retains the pentameric scaffold while exposing mCRP-like antigenic epitopes on its surface. It can bind C1q, activate the classical complement pathway, and promote leukocyte recruitment when associated with cell-derived microvesicles. pCRP* can subsequently dissociate into mCRP and therefore represents an intermediate in the local conformational activation of CRP rather than a separate parallel pathway (11).

Accordingly, the currently supported sequence is pCRP → pCRP* → mCRP. Membrane composition, acidic pH, oxidative conditions, calcium availability, and interactions with activated or damaged cells may influence this process. However, the relative abundance, kinetics, and reversibility of these conformational states in human cardiovascular tissues remain incompletely characterized (11, 30).

### 2.2 Synthesis regulation and expression characteristics of CRP

CRP synthesis exhibits both tissue specificity and inflammation dependence. The liver is the primary site of production. Under physiological conditions, baseline serum CRP levels are very low, typically ranging from 0 to 5 mg/L, although concentrations vary among individuals (31, 38). During inflammation, infection, tissue injury, or metabolic disturbance, a network of inflammatory cytokines initiates transcriptional and translational regulation of CRP. Interleukin-6 (IL-6) serves as the principal upstream signal for hepatic CRP synthesis. It activates the Janus kinase-signal transducer and activator of transcription 3 (JAK-STAT3) pathway, thereby promoting CRP transcription. Interleukin-1β (IL-1β) further enhances this response through complementary transcriptional pathways, including nuclear factor-κB (NF-κB) signaling (38). Tumor necrosis factor-α (TNF-α) and other inflammatory mediators can synergistically amplify the effects of IL-6, although IL-6 remains the dominant hepatic stimulus. Consequently, serum CRP levels rise within 6-12 h after an inflammatory stimulus, peak at 24-48 h, and decline rapidly upon resolution of inflammation, demonstrating sensitive inflammatory response kinetics (39). CRP released from the liver enters the circulation predominantly as soluble pCRP; hs-CRP refers to the analytical sensitivity of the assay and not to a distinct molecular form of CRP.

In addition to the classical hepatic pathway, CRP expression has been reported in vascular smooth muscle cells and endothelial cells under selected experimental conditions. CRP messenger RNA and protein have also been detected in some human atherosclerotic specimens (38, 40). However, other tissue studies found little CRP messenger RNA in vascular lesions and suggested that most plaque-associated CRP was derived from the circulation (41). Thus, the quantitative contribution of extrahepatic synthesis to vascular CRP deposition remains uncertain, and the liver is considered the predominant source of circulating CRP. Regardless of its source, pCRP deposited from the circulation can bind to damaged cellular membranes or microvesicles and undergo local conformational transition to pCRP* and mCRP. This mechanism provides a plausible link between circulating CRP and the accumulation of conformationally altered CRP within injured vascular tissue (11, 42).

Furthermore, baseline CRP expression levels show marked interindividual heterogeneity regulated by genetic polymorphisms, age, sex, genetic ancestry, and metabolic status (43, 44). Single-nucleotide polymorphisms within and around the CRP locus can influence circulating CRP concentrations. However, genetic effects on CRP concentrations should not be interpreted as evidence that CRP itself alters cardiovascular susceptibility (44, 45). In addition, factors such as estrogen levels, obesity, dysregulated glucose and lipid metabolism, and smoking can modulate CRP expression. These determinants should therefore be considered when interpreting hs-CRP concentrations across individuals and populations (43, 46).

### 2.3 Core biological functions of CRP and recent research advances

Based on the distinct features of its conformational forms, CRP exhibits context- and conformation-dependent biological functions. Under physiological homeostasis, it primarily supports protective immune regulation, whereas in pathological inflammatory states conformationally altered CRP can acquire proinflammatory activities in experimental systems (30, 47). Under physiological conditions, pCRP can recognize pathogens, damaged cells, and toxic macromolecules and facilitate their clearance. When bound to appropriate ligands, it activates the classical complement pathway to mediate immune defense (12, 34). Recent studies have reported that pCRP can bind damage-associated molecules such as β-amyloid fibrils and toxic histones, thereby limiting their associated cytotoxicity and tissue injury. These findings support a potential role for pCRP in recognition and clearance during inflammatory stress (34, 35).

In pathological conditions, experimental studies have attributed several vascular effects to conformationally altered CRP. Tissue-associated mCRP can impair endothelial barrier function by promoting oxidative stress, endothelial activation, and apoptosis. It can also influence vascular smooth muscle cell proliferation, migration, and phenotypic switching and can enhance leukocyte recruitment and macrophage inflammatory responses. In parallel, microvesicle-bound pCRP* can bind C1q and activate the classical complement pathway, thereby amplifying inflammation at sites of tissue injury (11, 12, 24, 42). In addition, experimental exposure to CRP preparations has been associated with cardiomyocyte mitochondrial fission through the extracellular signal-regulated kinase 1/2 (ERK1/2)–Yes-associated protein (YAP) signaling pathway (48). Because the conformational composition of the CRP preparation was not established in that study, this effect cannot be assigned specifically to mCRP.

Recent research has moved beyond the study of CRP as an isolated molecule to focus on its interactive regulatory networks with metabolic and immune molecules. *In vitro* studies using CRP preparations have reported interactions with low-density lipoprotein (LDL), oxidized lipids, and high-density lipoprotein (HDL), suggesting that the biological consequences of these interactions depend on ligand exposure and CRP conformation (49). On the other hand, novel composite molecular markers based on CRP continue to be identified. Indices such as the hs-CRP to high-density lipoprotein cholesterol ratio and the CRP-triglyceride-glucose index integrate inflammatory activation and metabolic disturbance. Compared with CRP alone, these composites may improve the identification of subclinical atherosclerosis and residual cardiovascular risk, providing potential adjunctive tools for early detection of occult CVD (14, 50).

Furthermore, research on novel molecular phenotypes, including CRP autoantibodies, has further enriched the molecular regulatory framework of CRP. Circulating autoantibodies against mCRP have also been investigated as a distinct immune readout. One study reported lower anti-mCRP autoantibody levels in patients with acute coronary syndrome than in patients with stable angina and healthy controls (51). Their mechanistic and prognostic significance remains uncertain.

### 2.4 Measurement of CRP conformational forms

Routine clinical CRP assays quantify circulating CRP by immunochemical methods, whereas hs-CRP assays use analytically more sensitive platforms to measure lower concentrations of the same analyte. Neither conventional CRP nor hs-CRP assays are designed to distinguish pCRP, pCRP*, and mCRP. Because soluble circulating CRP is predominantly pentameric, routine measurements largely reflect soluble pCRP and may not capture cell-, platelet-, or microvesicle-associated conformationally altered CRP (52).

Conformation-specific CRP detection remains primarily a research application. Neoepitope-directed monoclonal antibodies have been used in sandwich enzyme-linked immunosorbent assays (ELISAs), immunoblotting, immunofluorescence, and immunohistochemistry to detect mCRP in plasma or tissue specimens (53, 54). Flow cytometry can identify pCRP- or mCRP-bearing circulating cells and extracellular vesicles and may therefore detect CRP pools not measured by routine soluble-protein assays (52). Several methodological limitations impede clinical translation. Antibodies directed against neoepitopes exposed after conformational rearrangement may recognize both pCRP* and mCRP, making the two forms difficult to distinguish immunologically (11). Sample collection, calcium chelation, pH, temperature, freeze-thaw cycles, contact with artificial surfaces, and membrane or lipid contamination may also induce or alter CRP dissociation ex vivo. At present, no internationally standardized clinical assay, reference material, or validated reference interval is available for mCRP or pCRP*. Consequently, results obtained using research assays should be interpreted in relation to the antibodies, sample-processing procedures, and molecular fractions examined.

### 2.5 Causality debate: biomarker or pathogenic mediator?

Although research on the molecular structure and biological functions of CRP has advanced considerably, whether CRP is a causal mediator of CVD or principally a biomarker of underlying inflammation remains unresolved. Available evidence differs according to the CRP form examined, the experimental system used, and the cardiovascular outcome considered.

Prospective observational studies consistently associate elevated circulating CRP, particularly when measured by hs-CRP assays, with incident cardiovascular events, recurrent events, and mortality (18, 19, 55). These associations establish hs-CRP as a clinically informative marker of inflammatory risk but cannot independently determine causality because CRP concentrations are influenced by adiposity, smoking, metabolic dysfunction, infection, comorbid disease, and other inflammatory mediators. Moreover, hs-CRP assays do not identify the conformational form or tissue source of the measured protein.

Genetic studies provide evidence against a major causal effect of lifelong elevation in circulating CRP. Large Mendelian randomization analyses found that CRP-associated genetic variants altered circulating CRP concentrations without producing the corresponding increase in coronary heart disease expected from observational associations (8, 9, 56). These findings argue against soluble circulating CRP being a major independent cause of atherosclerotic cardiovascular events, although they do not directly evaluate locally generated pCRP* or mCRP.

Animal studies have also produced inconsistent results. Targeted deletion of CRP in Apoe-deficient and Ldlr-deficient mice did not reduce atherosclerotic lesion formation and, in some comparisons, was associated with larger lesions (57). In a mouse model with a more human-like lipoprotein profile, transgenic human CRP slowed rather than accelerated atherosclerosis (58). Administration of an engineered, conformationally altered CRP capable of binding atherogenic lipoproteins reduced aortic lesion burden in Ldlr-deficient mice (47). More recently, a study using Crp-deficient Apoe-deficient mice demonstrated a diet-dependent effect, with CRP deletion reducing atherosclerotic plaque burden under high-fat feeding but not under chow feeding (59). Together, these studies do not support a uniform effect of CRP on atherogenesis and indicate that the outcome depends on dietary and metabolic conditions, species biology, lipoprotein context, CRP conformation, and the experimental model.

By contrast, tissue and mechanistic studies provide evidence that conformationally altered CRP can act as a local inflammatory effector. Membrane-bound pCRP can transition to pCRP*, activate complement, recruit leukocytes, and subsequently dissociate into mCRP. In experimental systems, inhibition of membrane-associated conformational activation or dissociation-attenuated inflammatory injury (11, 42). These findings support the biological activity of pCRP* and mCRP at sites of tissue injury but do not establish that either form is necessary or sufficient to cause human CVD.

Evidence from intervention studies requires a similar distinction. In CANTOS, inhibition of interleukin-1β reduced recurrent cardiovascular events among patients with previous myocardial infarction and elevated hs-CRP, demonstrating that upstream inflammation is modifiable without directly targeting CRP (16). Reductions in hs-CRP during anti-inflammatory therapy therefore indicate suppression of inflammatory activity but do not demonstrate that CRP reduction mediates the clinical benefit. Direct CRP-targeting approaches remain less mature. Selective CRP apheresis has reduced circulating CRP in exploratory studies, whereas randomized trials have been initiated to determine its effects on myocardial injury and clinical outcomes (60, 61). Conformation-modulating small molecules and antibodies remain predominantly at the preclinical stage.

Taken together, evidence supports a differentiated interpretation: hs-CRP is an established biomarker of systemic inflammation and cardiovascular risk; a direct causal role for circulating pCRP in atherosclerotic CVD has not been established; and pCRP* and mCRP remain plausible local biological effectors supported mainly by structural, cellular, tissue, and preclinical evidence. This framework should guide the interpretation of CRP throughout the disease-specific sections of this review.

Future research should prioritize standardized conformation-specific assays, experimental models that more closely reproduce human CRP, complement, and lipoprotein biology, and interventions that selectively inhibit pathological conformational transitions while preserving the physiological functions of pCRP. Prospective studies combining circulating hs-CRP with tissue- or vesicle-associated pCRP* and mCRP measurements will be necessary to determine whether conformation-specific assessment improves cardiovascular risk stratification or therapeutic selection (30, 62).

**Figure 2:**
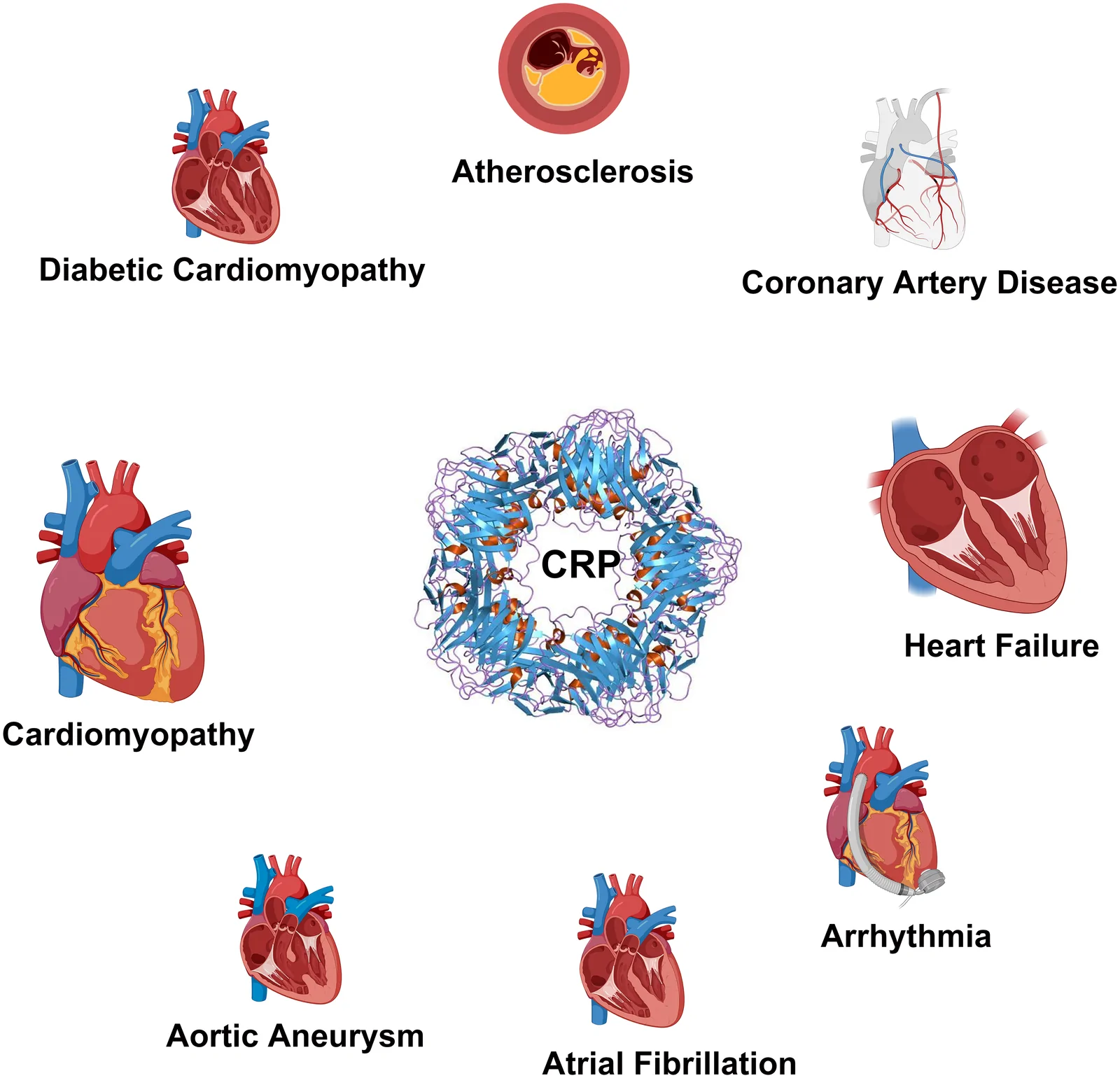
Overview of cardiovascular conditions discussed in relation to C-reactive protein. The schematic places CRP at the center and summarizes the principal cardiovascular conditions covered in this review, including atherosclerosis, coronary artery disease, heart failure, arrhythmia, atrial fibrillation, aortic aneurysm, cardiomyopathy, and diabetic cardiomyopathy. The central CRP representation serves as an organizational link between the subsequent disease-specific sections.

**Figure 2** provides a schematic overview of the principal cardiovascular conditions discussed in the following sections.

## 3 CRP in coronary artery disease

Coronary artery disease (CAD) is a chronic inflammatory vascular disease characterized by the formation, progression, and rupture of atherosclerotic plaques in the coronary arteries, along with secondary thrombosis. Inflammatory and immune responses contribute to plaque progression, destabilization, and subsequent thrombotic complications (63, 64).

### 3.1 Clinical significance of hs-CRP in CAD

CRP is currently the most extensively studied and widely applied inflammatory biomarker in the CAD field. High-sensitivity CRP (hs-CRP) assays enable the detection of low-grade systemic inflammation. Elevated circulating hs-CRP is associated with incident CAD, greater coronary lesion burden, plaque instability, recurrent cardiovascular events, and cardiovascular mortality. Accordingly, hs-CRP has been incorporated into the assessment of inflammatory risk, disease severity, treatment response, and long-term prognosis (65, 66).

### 3.2 Conformation-dependent and metabolic mechanisms in CAD

Conformation-dependent activation provides a mechanistic link between circulating CRP and inflammation within atherosclerotic plaques. Circulating pCRP can bind to phosphocholine-containing membranes on activated platelets and cell-derived microvesicles, leading to the sequential transition pCRP → pCRP* → mCRP. This structural rearrangement exposes proinflammatory binding sites that are largely inaccessible in circulating pCRP (11, 36).

Within atherosclerotic lesions, experimental and tissue-based studies indicate that pCRP* and mCRP can enhance complement activation, leukocyte recruitment, endothelial activation, and platelet–leukocyte interactions. Deposited mCRP has been detected in human atherosclerotic plaques in association with macrophages and activated platelets. These localized effects may intensify plaque inflammation, impair endothelial integrity, and facilitate thrombus formation following plaque disruption (42).

Recent genetic evidence also indicates that the relationship between CRP and atherosclerosis is influenced by the metabolic environment. In Apoe-deficient mice, Crp deletion did not alter atherosclerosis during chow feeding but significantly reduced plaque burden during high-fat feeding. The effect was attributed primarily to a liver-to-vessel axis in which CRP upregulated cell death-inducing DFFA-like effector A (Cidea), enhanced hepatic lipid-droplet accumulation, aggravated hyperlipidemia, and thereby promoted atherosclerosis, rather than to a direct increase in vascular inflammation. The same study found that the effects of atorvastatin on high-fat diet-induced hepatic and vascular abnormalities were strongly influenced by CRP status. These context-dependent findings may help reconcile earlier discordant results obtained in conventional chow-fed mouse models and suggest that the contribution of CRP to atherogenesis varies with the metabolic environment (59). This liver-to-vessel pathway connects CRP with hepatic lipid handling, circulating lipid burden, and subsequent plaque development under high-fat dietary conditions.

### 3.3 Genetic regulation of CRP in CAD

Genetic variation contributes to interindividual differences in circulating CRP levels. Multiple population studies have shown that polymorphisms at CRP gene loci (CRP −757 A/G, CRP −717 T/C) and in the upstream IL-6 gene (−572 C/G) are associated with baseline CRP levels, although their relationships with CAD risk and severity vary across populations (45, 67). Meta-analytic evidence similarly indicates substantial heterogeneity among CRP variants, ethnic groups, and clinical phenotypes (68).

Lipid metabolism-related gene polymorphisms, including variants involving apolipoproteins, paraoxonase-1, and ATP-binding cassette transporter A1, may influence circulating inflammatory markers through alterations in lipid metabolism and oxidative stress (69, 70). These genetic interactions illustrate the close relationship among lipid metabolism, systemic inflammation, and CAD susceptibility.

### 3.4 Clinical assessment and prognostic stratification

hs-CRP complements clinical and imaging assessment by providing information on systemic inflammatory activity. It may be particularly useful for identifying residual inflammatory risk in patients with established CAD and for characterizing inflammatory activation in non-obstructive CAD (71, 72).

Serum hs-CRP levels are associated with coronary lesion severity, extent, and stability, and positive correlations have been reported with SYNTAX and Gensini scores. Higher hs-CRP levels are also associated with multivessel disease, severe stenosis, and vulnerable plaques (73, 74). The CRP-to-albumin (CRP/ALB) ratio, a composite marker of inflammation and nutrition, has also been associated with coronary lesion burden and complexity (75, 76). Combining hs-CRP with metabolic indices such as the atherogenic index of plasma and triglyceride-glucose index may further improve the identification of patients with a high atherosclerotic burden (77, 78).

In stable CAD, elevated baseline hs-CRP is associated with an increased risk of subsequent myocardial infarction and cardiovascular death, including among patients who achieve recommended lipid targets (79, 80). Elevated hs-CRP measured before or after percutaneous coronary intervention (PCI) is also associated with in-stent restenosis and long-term major adverse cardiovascular events (81, 82).

The prognostic value of hs-CRP may be particularly relevant in patients with chronic kidney disease or renal insufficiency (83, 84). Combining hs-CRP with N-terminal pro-B-type natriuretic peptide may provide additional information for predicting cardiovascular events and clinical restenosis after coronary angioplasty (85). In population-based risk assessment, the combination of hs-CRP and homocysteine has also been associated with an increased estimated risk of CAD (86).

### 3.5 Anti-inflammatory treatment and hs-CRP monitoring

hs-CRP is commonly used to assess residual inflammatory risk and monitor changes in inflammatory activity during CAD treatment. Statins lower hs-CRP levels beyond lipid-lowering effects through several anti-inflammatory pathways. High-intensity atorvastatin produces stronger anti-inflammatory effects and may improve collateral circulation while slowing plaque progression (87, 88). Patients who maintain low hs-CRP after statin therapy generally have a lower risk of subsequent cardiovascular events, supporting the use of hs-CRP in treatment-response and residual-risk assessment (89).

CANTOS randomized 10,061 patients with previous myocardial infarction and hs-CRP concentrations of at least 2 mg/L to placebo or canakinumab, a monoclonal antibody targeting interleukin-1β. Canakinumab administered at 150 mg every 3 months produced an approximately 15% relative reduction in the primary composite cardiovascular endpoint without reducing low-density lipoprotein cholesterol. However, treatment was associated with a higher incidence of fatal infection or sepsis and did not reduce all-cause mortality (16). CANTOS established upstream interleukin-1β inhibition as a potential strategy for patients with residual inflammatory risk. In this trial, hs-CRP was used to select patients with persistent inflammatory activity and to monitor suppression of the interleukin-1β-interleukin-6-CRP pathway.

Colchicine has been evaluated as an upstream anti-inflammatory therapy in several cardiovascular outcome trials. The Colchicine Cardiovascular Outcomes Trial (COLCOT) and the second Low-Dose Colchicine trial (LoDoCo2) reported reductions in ischemic cardiovascular events among patients with recent myocardial infarction and chronic CAD, respectively (90, 91). By contrast, CLEAR (Colchicine and Spironolactone in Patients with Myocardial Infarction) found no significant reduction in its primary composite cardiovascular endpoint when colchicine was initiated soon after acute myocardial infarction (92). Differences in patient selection, treatment timing, adherence, and clinical setting may contribute to these variable findings.

Non-pharmacologic interventions, including exercise-based cardiac rehabilitation, weight management, and continuous positive airway pressure, can also lower hs-CRP and improve cardiovascular risk profiles, supporting hs-CRP as a practical marker of changes in systemic inflammatory activity (93, 94). Direct CRP-targeted approaches, including CRP apheresis and inhibitors of CRP conformational activation, remain at the experimental or early clinical stage (62).

### 3.6 Current challenges and future research directions

Despite considerable progress, several important challenges remain. The clinical interpretation of hs-CRP requires consideration of systemic conditions such as infection or autoimmune disease. Distinguishing CAD-specific vascular inflammation from non-specific inflammation remains a key challenge (95). Although tissue deposition of pCRP* and mCRP has been demonstrated experimentally, the clinical relationship among local conformational activation, circulating hs-CRP, plaque characteristics, and cardiovascular events remains insufficiently characterized. Conformation-specific CRP assays and direct CRP-targeted treatments are also not yet available for routine clinical use. Population ancestry, sex, metabolic status, and comorbidities further contribute to variation in hs-CRP distribution and influence the interpretation of clinical thresholds (68, 96).

Future studies should evaluate the incremental value of serial hs-CRP measurement when combined with established clinical risk scores, metabolic markers, and coronary imaging. The development of standardized conformation-specific assays may help connect circulating inflammatory risk with local plaque biology. Prospective studies of CRP removal or inhibition will further clarify the therapeutic potential of targeting CRP in CAD.

## 4 CRP in heart failure

Heart failure (HF) represents the end stage of various cardiovascular diseases. It affects more than 64 million people worldwide and carries a 5-year mortality rate of approximately 50%, making it one of the most significant public health challenges of the 21st century (97, 98). While traditional understanding has centered on neurohormonal activation as the primary driver of HF, accumulating evidence over the past two decades indicates that chronic low-grade inflammation is common across the HF spectrum and is associated with myocardial injury, ventricular remodeling, functional deterioration, and adverse outcomes (98, 99). Circulating high-sensitivity C-reactive protein (hs-CRP) provides a clinically accessible measure of systemic inflammatory activity and has been associated with HF severity, hospitalization, and mortality (100, 101). In recent years, advances in understanding the mechanisms of heart failure with preserved ejection fraction (HFpEF), together with the emergence of novel anti-inflammatory agents and multimarker strategies, have brought renewed attention to CRP as a marker of inflammatory and metabolic phenotypes in HF.

### 4.1 Biological and subtype-specific context of CRP in HF

#### 4.1.1 Experimental evidence for myocardial injury and remodeling

Binding of pCRP to phosphocholine-containing membranes at sites of tissue injury can promote its conformational transition to pCRP* and mCRP. These altered forms expose functional sites involved in C1q binding and classical complement activation. Zimmermann et al. observed co-localization of CRP and C5b-9 in myocardial tissue from patients with non-ischemic HF, with the degree of co-localization correlating positively with macrophage infiltration, linking myocardial CRP deposition with local complement-associated inflammation (20).

Experimental studies have linked CRP exposure to cardiomyocyte inflammatory signaling, oxidative stress, altered calcium handling, and apoptosis. The magnitude and direction of these effects depend on the experimental system and the CRP preparation used (97, 98).

CRP-related inflammatory signaling has also been associated experimentally with cardiac fibroblast activation, myofibroblast differentiation, transforming growth factor-β signaling, and increased extracellular matrix deposition (97, 98). Myocardial fibrosis is a major contributor to ventricular stiffness and diastolic dysfunction and represents a core pathological feature of HFpEF (102).

Inflammatory activation in HF is accompanied by myocardial recruitment of monocytes, macrophages, and neutrophils. Tissue-associated CRP and activated innate immune cells may interact locally with complement and cytokine pathways, thereby contributing to sustained myocardial inflammation and remodeling (103, 104).

#### 4.1.2 NETs and innate immune activation in HF

Neutrophil extracellular traps (NETs) are web-like structures composed of DNA, histones, and granule proteins released by neutrophils upon stimulation. Although they serve as a defense mechanism against pathogens, excessive NET formation can cause tissue damage and sustained inflammation. Vulesevic et al. reported that CRP exposure activated neutrophils via the FcγRIIb receptor to induce NETosis, with this effect being more pronounced in HF patients with concomitant diabetes (104). Circulating markers of NET formation, including citrullinated histone H3 and DNA-neutrophil elastase complexes, are elevated in patients with HF and have been associated with hs-CRP, diabetes, and disease severity (103). NET-derived histones, proteases, and extracellular DNA may contribute to endothelial injury, thrombosis, myocardial inflammation, and fibrosis.

#### 4.1.3 Obesity-related inflammatory phenotype in HFpEF

Heart failure is a highly heterogeneous syndrome. Based on left ventricular ejection fraction (LVEF), it is classified into heart failure with reduced ejection fraction (HFrEF, LVEF <40%), heart failure with mildly reduced ejection fraction (HFmrEF, LVEF 40%-49%), and heart failure with preserved ejection fraction (HFpEF, LVEF ≥50%). HFpEF accounts for more than 50% of HF cases and encompasses several clinical phenotypes with distinct hemodynamic, metabolic, and inflammatory features. Comorbidity-associated systemic inflammation is particularly prominent in obesity-related HFpEF (102, 105).

Obesity, type 2 diabetes, and metabolic syndrome are common components of HFpEF. These comorbidities can cause adipose tissue dysfunction, leading to excessive secretion of IL-6, TNF-α, and other cytokines that stimulate hepatic CRP production and generate systemic chronic low-grade inflammation (106–108). Adipose-tissue inflammation, endothelial activation, and reduced nitric oxide (NO) bioavailability contribute to coronary microvascular dysfunction, myocardial hypoperfusion, cardiomyocyte hypertrophy, interstitial fibrosis, and impaired diastolic function. In this setting, elevated hs-CRP reflects the combined inflammatory burden generated by adipose tissue dysfunction and associated metabolic comorbidities (102, 105). Sabbah et al. used cluster analysis to identify three HFpEF phenotypes, among which the obese-inflammatory phenotype exhibited the highest hs-CRP levels and the worst prognosis, identifying a clinically distinct inflammation-enriched HFpEF subgroup (102).

Consistent with this model, circulating inflammatory markers, including CRP, are positively associated with myocardial extracellular volume measured by cardiac magnetic resonance, linking systemic inflammation to diffuse myocardial fibrosis at the population level (108). Together, these observations support an obesity-associated inflammatory HFpEF phenotype in which hs-CRP reflects the combined burden of adipose-tissue inflammation, microvascular dysfunction, and myocardial remodeling.

In contrast, the inflammatory response in HFrEF is more closely linked to direct myocardial injury (e.g., myocardial infarction or myocarditis), with higher circulating CRP concentrations accompanying post-infarction inflammatory repair and ventricular remodeling (109–111). However, recent studies indicate that even in HFrEF, coexisting metabolic comorbidities such as obesity and diabetes can elevate CRP levels and are associated with worse outcomes, suggesting overlapping inflammatory mechanisms across HF subtypes (111).

#### 4.1.4 HF-specific genetic evidence

Polymorphisms in the CRP gene can influence baseline CRP expression levels and have been examined in relation to HF outcomes. Opielak et al. reported that the CRP rs2794521 polymorphism was significantly associated with survival in patients with chronic HF and depression; carriers of the T allele had higher mortality (112). Kittel-Schneider et al. further showed that CRP gene variants were associated not only with mortality but also with depression severity in HF patients, highlighting a potential genetic relationship among CRP expression, HF prognosis, and depressive symptoms (113).

Observational studies consistently show that elevated CRP levels are associated with increased HF incidence and adverse prognosis (114, 115). However, Mendelian randomization (MR) studies have yielded inconsistent results. Remmelzwaal et al. found no causal relationship between CRP and incident HF in a two-sample MR analysis (116), whereas Habibi et al. reported that genetically predicted higher CRP levels were associated with increased HF risk (117). This heterogeneity may stem from differences in genetic instruments, study populations, and HF subtype composition, particularly the distinct pathophysiologies of HFpEF and HFrEF (116, 117). HF-specific genetic evidence therefore indicates substantial heterogeneity across populations and HF phenotypes, consistent with the broader causality framework discussed in Section 2.5.

### 4.2 Clinical associations and risk stratification

#### 4.2.1 hs-CRP across HF phenotypes and disease severity

High-sensitivity CRP (hs-CRP) provides useful adjunctive information about systemic inflammatory activity in HF. In patients with acute HF, hs-CRP levels on admission are significantly elevated and correlate positively with New York Heart Association (NYHA) functional class (118, 119). In chronic HF, hs-CRP levels are also higher than in healthy individuals and tend to rise with increasing disease severity (120, 121). In acute HF, interpretation of hs-CRP is influenced by infection and other systemic inflammatory conditions. Elevated CRP was associated with adverse prognosis more consistently in patients without concomitant infection (122, 123).

The distribution of CRP also differs across HF subtypes. Meta-analyses indicate that hs-CRP levels are generally higher in patients with heart failure with preserved ejection fraction (HFpEF) than in those with heart failure with reduced ejection fraction (HFrEF), in part reflecting the greater prevalence of obesity, diabetes, and other inflammatory comorbidities in HFpEF (124). Variation in nutritional status, hemodynamic severity, renal function, and comorbidity burden contributes to the overlap in hs-CRP concentrations across HF phenotypes (125).

#### 4.2.2 Multimarker and composite inflammatory indices

Multimarker strategies combine hs-CRP with biomarkers that reflect myocardial stress, injury, fibrosis, or systemic disease. Panels incorporating N-terminal pro-B-type natriuretic peptide (NT-proBNP), troponin, soluble suppression of tumorigenicity 2 (sST2), growth differentiation factor-15 (GDF-15), and galectin-3 have been evaluated for HF phenotyping and risk stratification (126, 127).

Dupuy et al. reported that the combination of sST2 and CRP provided superior prognostic information in chronic HF compared with either marker alone (128). Sinning et al. developed a composite index incorporating CRP, GDF-15, sST2, and NT-proBNP that differentiated HF phenotypes and provided prognostic information (129). Combining CRP with echocardiographic parameters has also been shown to enhance clinical assessment in chronic HF (130).

Composite indices based on routinely available measurements have also been investigated. The C-reactive protein-albumin-lymphocyte (CALLY) index integrates inflammation, nutritional status, and lymphocyte count, whereas the CRP-to-albumin ratio, HDL-C-to-CRP ratio, and CRP-triglyceride-glucose index combine inflammatory information with nutritional or metabolic variables. These indices have shown associations with mortality, hospitalization, and length of stay in observational HF cohorts, particularly among elderly, metabolically impaired, or malnourished patients (131–135).

#### 4.2.3 Prognostic and dynamic value of hs-CRP

Multiple prospective cohort studies and meta-analyses have consistently demonstrated that hs-CRP is an independent predictor of all-cause mortality, cardiovascular mortality, and heart failure rehospitalization in patients with HF (115, 135, 136). In chronic HF, each one-standard-deviation increase in hs-CRP is associated with a 20%-30% higher risk of all-cause death (136). The Valsartan Heart Failure Trial (Val-HeFT) was the first large-scale clinical study to identify baseline CRP as an independent predictor of mortality and rehospitalization in chronic HF, independent of left ventricular ejection fraction (LVEF) and New York Heart Association (NYHA) class (135).

In acute HF, the prognostic value of CRP has also been widely validated. Recent studies suggest that discharge CRP levels may have greater predictive utility than admission levels (137). Nishimoto et al. reported that patients with acute decompensated HF who had hs-CRP >5 mg/L at discharge had a 2.3-fold higher 1-year mortality compared with those whose hs-CRP was ≤5 mg/L (137). Moreover, dynamic changes in CRP appear more informative than single measurements: patients whose CRP levels declined after treatment had significantly better outcomes than those with persistently elevated CRP (138, 139).

The prognostic value of CRP and related composite indices is particularly pronounced in certain high-risk HF subgroups. In elderly patients with HF, who often have multiple comorbidities and more prominent inflammation and malnutrition, the CALLY index has emerged as a potential indicator of long-term risk (140).

In frail patients with HF, inflammation serves as a shared pathological pathway between frailty and HF progression. Meta-analyses have shown that CRP levels are significantly higher in frail HF patients than in non-frail patients and correlate positively with frailty severity (141–143). Ribeiro et al. further identified hs-CRP as an independent predictor of incident frailty in HF patients, suggesting its potential role in identifying individuals at high risk for frailty-related complications (141).

Adding CRP to established HF prognostic models can improve their predictive performance. Wussler et al. demonstrated that incorporating hs-CRP into the Multiple Estimation of Risk Based on the Emergency Department Spanish Score in Patients With Acute Heart Failure (MEESSI-AHF) increased the C-statistic from 0.79 to 0.83 for the prediction of 30-day mortality (143). These findings support the evaluation of hs-CRP within multimarker and clinical risk models rather than as an isolated prognostic measure (128, 129).

### 4.3 Treatment-associated changes in CRP

#### 4.3.1 Established HF therapies and SGLT2 inhibitors

Traditional neurohormonal antagonists not only improve clinical symptoms and outcomes in patients with heart failure (HF) but also exert modest anti-inflammatory effects that can lower CRP levels. In the Val-HeFT trial, valsartan significantly reduced CRP levels in patients with chronic HF, and the magnitude of CRP reduction correlated with clinical benefit (135). β-blockers have been shown to lower hs-CRP levels in elderly patients with HF after 12 weeks of treatment, accompanied by improvements in 6-minute walk distance and cardiac function (144). Changes in hs-CRP during these treatments reflect broader neurohormonal and inflammatory modulation.

Sodium-glucose cotransporter 2 (SGLT2) inhibitors reduce HF hospitalization and cardiovascular events through broad cardiorenal, metabolic, and hemodynamic effects. In a biomarker analysis of the Dapagliflozin and Prevention of Adverse Outcomes in Heart Failure trial (DAPA-HF), higher baseline IL-6 and hs-CRP concentrations were associated with worse outcomes, whereas the clinical benefit of dapagliflozin was consistent across baseline inflammatory-marker strata. Dapagliflozin did not significantly reduce IL-6 or hs-CRP compared with placebo at 12 months (145). Smaller real-world studies have reported reductions in circulating inflammatory markers during SGLT2 inhibitor treatment (146), indicating variable biomarker responses across clinical settings.

#### 4.3.2 Obesity-targeted pharmacotherapy in HFpEF

The STEP-HFpEF trial randomized 529 patients with obesity-related HFpEF to once-weekly semaglutide 2.4 mg or placebo for 52 weeks. Semaglutide produced greater improvements in the Kansas City Cardiomyopathy Questionnaire Clinical Summary Score and 6-minute walk distance, greater weight loss, and a larger reduction in CRP; mean CRP changed by −43.5% with semaglutide versus −7.3% with placebo (147). The STEP-HFpEF DM trial and pooled analyses of the two trials extended these findings to patients with type 2 diabetes (148). In a dedicated analysis of inflammation in the STEP-HFpEF program, semaglutide improved symptoms, physical limitations, and exercise function across baseline CRP categories and reduced CRP substantially relative to placebo. In this setting, hs-CRP served as a treatment-responsive marker of the inflammatory component of obesity-related HFpEF (106).

Consistent evidence has emerged from the SUMMIT trial, in which 731 patients with HFpEF and obesity were randomized to tirzepatide or placebo. Tirzepatide reduced the composite risk of cardiovascular death or worsening heart failure, improved health status, and lowered hs-CRP at 52 weeks (149). Semaglutide, a GLP-1 receptor agonist, and tirzepatide, a dual glucose-dependent insulinotropic polypeptide/GLP-1 receptor agonist, modify hs-CRP indirectly through weight reduction, metabolic improvement, and attenuation of systemic inflammation. These trials support phenotype-directed treatment of obesity-related HFpEF, with hs-CRP providing a readily measurable indicator of treatment-associated inflammatory change.

#### 4.3.3 Inflammation-directed therapy

Given the involvement of inflammation in HF, therapies targeting inflammatory pathways have long been an area of active investigation. However, early trials of anti-inflammatory agents, such as anti-TNF-α therapies, failed to demonstrate clinical benefit, highlighting the complexity of inflammatory mechanisms in HF and the need for more precise strategies (150).

In the 12-patient Diastolic Heart Failure Anakinra Response Trial (D-HART) pilot study, 14 days of anakinra reduced CRP and produced a modest improvement in peak oxygen consumption (151). In the subsequent D-HART2 trial, 31 patients with HFpEF and CRP >2 mg/L received anakinra or placebo for 12 weeks. Anakinra reduced systemic inflammatory activity but did not improve the primary endpoints of peak oxygen consumption or ventilatory efficiency (152). Evidence from randomized anakinra studies in HF remains based on relatively small populations and short follow-up periods (153). An observational registry analysis found no significant reduction in 30-day mortality or postdischarge events with intravenous corticosteroid therapy, although treatment estimates varied according to baseline CRP concentration (154).

Current evidence suggests that the effects of anti-inflammatory therapy may vary among HF phenotypes. Prospective studies are needed to determine whether CRP-based inflammatory phenotyping improves patient selection or clinical outcomes during pathway-specific anti-inflammatory treatment (136, 155).

#### 4.3.4 Non-pharmacological interventions

Non-pharmacological interventions also play a meaningful role in HF management by lowering inflammation and improving outcomes. Systematic reviews indicate that regular aerobic exercise and resistance training can reduce CRP levels in patients with chronic HF while enhancing exercise capacity and quality of life (156). Nutritional interventions, including the Mediterranean diet and ω-3 fatty acid supplementation, have been associated with lower inflammatory markers; meta-analyses show that ω-3 supplementation can reduce both CRP and TNF-α levels in HF patients (157). Multidisciplinary care models centered on HF specialists optimize medication regimens and lifestyle modifications and are associated with better prognosis (158).

### 4.4 Challenges and future perspectives

Despite considerable progress in research on CRP in heart failure (HF), several important challenges remain. Circulating hs-CRP is an established marker of systemic inflammatory activity and prognosis, whereas the clinical relevance of myocardial pCRP*, mCRP, and local CRP deposition remains less well characterized. Variation in HF phenotype, comorbidity burden, sampling time, infection status, and hs-CRP thresholds also contributes to heterogeneity among clinical studies.

Future studies should determine whether serial hs-CRP measurement provides clinically meaningful information beyond established HF risk scores, natriuretic peptides, and imaging. Conformation-specific assays may help define the relationship between circulating inflammatory activity and local myocardial CRP deposition. Prospective trials should also identify the HF phenotypes most likely to benefit from upstream anti-inflammatory treatment or direct modification of CRP.

In summary, CRP is one of the most extensively studied inflammatory biomarkers in HF. Circulating hs-CRP provides clinically relevant information on systemic inflammatory burden and prognosis, whereas experimental evidence concerning tissue-associated CRP conformations offers a complementary framework for studying myocardial inflammation. The obesity-related HFpEF phenotype is particularly informative because metabolic improvement with semaglutide and tirzepatide is accompanied by substantial reductions in hs-CRP and improved clinical status.

## 5 CRP in cardiac arrhythmias

Cardiac arrhythmias are among the most common clinical manifestations of cardiovascular disease and contribute substantially to cardiovascular morbidity and mortality. Accumulating evidence indicates that inflammatory responses play an important role in the initiation, progression, and maintenance of arrhythmias. C-reactive protein (CRP), a classic marker of the acute-phase response, has been extensively investigated as a circulating marker of inflammation in this setting. Since Chung et al. first reported elevated serum CRP levels in patients with atrial fibrillation (AF) in 2001 (159), subsequent studies have examined its associations with AF occurrence, recurrence, and adverse outcomes, together with its potential involvement in atrial remodeling. The available evidence is strongest for AF, whereas data concerning ventricular arrhythmias and conduction disorders remain comparatively limited.

### 5.1 CRP in atrial fibrillation: from epidemiological associations to mechanistic exploration

Atrial fibrillation is the most common sustained arrhythmia in clinical practice. Approximately 59.7 million people were living with AF or atrial flutter worldwide in 2019, and these conditions substantially increase the risks of stroke, heart failure, and death (160). The relationship between CRP and AF is one of the most extensively studied areas in the field of inflammation and arrhythmias.

#### 5.1.1 Epidemiological evidence

Large population-based cohort studies have consistently shown that elevated circulating CRP levels are associated with an increased risk of incident AF. In the UK Biobank study involving 478,524 participants, Yang et al. found that each one-standard-deviation increase in CRP was associated with a 12% higher risk of AF (HR = 1.12, 95% CI 1.08-1.16) (161). A 2026 *post-hoc* analysis linking serial CRP measurements with continuous rhythm monitoring also demonstrated a temporal dose–response relationship. Each doubling of CRP was associated with 36% higher odds of daily AF lasting at least 1 h (adjusted OR = 1.36, 95% CI 1.26-1.47), whereas CRP concentrations of >10 to 50 mg/L and >50 mg/L were associated with approximately 3.5-fold and 6-fold higher odds of AF, respectively, compared with concentrations ≤3 mg/L (162). This association has been reported in populations with hypertension, metabolic syndrome, and coronary artery disease (163–165).

CRP levels are associated not only with the development of AF but also with its severity and type. Several studies have reported higher circulating CRP or hs-CRP levels in patients with persistent or permanent AF compared with those with paroxysmal AF (165, 166). In patients with rheumatic mitral stenosis, baseline hs-CRP was an independent predictor of future atrial arrhythmias in those initially in sinus rhythm (167). Elevated CRP is also linked to worse prognosis in patients with AF. A meta-analysis of 23 studies showed that higher CRP levels in AF were associated with increased risks of all-cause mortality (HR = 1.48, 95% CI 1.23-1.78), stroke (HR = 1.52, 95% CI 1.21-1.91), and major adverse cardiovascular events (HR = 1.63, 95% CI 1.32-2.01) (168). A 2026 nationwide registry study further confirmed that patients with AF with CRP >3 mg/L had a 1.78-fold higher risk of heart failure compared with those whose CRP was ≤3 mg/L (HR = 1.78, 95% CI 1.51-2.10, P < 0.001) (169).

#### 5.1.2 CRP-associated inflammation and atrial remodeling

Experimental studies have investigated the effects of CRP-associated inflammatory signaling on atrial cells. In HL-1 atrial cardiomyocytes, exposure to recombinant human CRP activated the Toll-like receptor 4 (TLR4)/nuclear factor-κB (NF-κB)/transforming growth factor-β (TGF-β) pathway, increased IL-6 and TGF-β1-related signaling, inhibited cell proliferation, and promoted apoptosis (170). These cellular responses provide a potential link between CRP-associated inflammation and the development of an arrhythmogenic atrial substrate.

Atrial fibrosis is an important substrate for AF initiation and maintenance. Activation of TGF-β-related signaling in CRP-exposed HL-1 cells is consistent with a potential connection between inflammatory signaling and profibrotic remodeling (170). In clinical studies, circulating CRP has been associated with collagen-remodeling biomarkers, P-wave dispersion, and left atrial enlargement (171, 172). Together, these findings link systemic inflammatory activity with electrical and structural features of the AF substrate.

Systemic inflammation may also interact with autonomic and metabolic factors that influence AF susceptibility. In patients with obstructive sleep apnea, continuous positive airway pressure therapy was associated with lower CRP levels and fewer arrhythmic events (173).

#### 5.1.3 Risk stratification for postoperative AF and post-ablation recurrence

CRP is a readily available biomarker that has been evaluated for risk stratification, particularly for postoperative atrial fibrillation and recurrence after catheter ablation. Postoperative atrial fibrillation is a common complication after cardiac surgery, occurring in 20%-40% of patients and associated with increased mortality and prolonged hospitalization. Multiple meta-analyses have shown that elevated preoperative and early postoperative CRP levels are associated with its occurrence (174, 175). Preoperative measurements reflect baseline inflammatory activity, whereas postoperative CRP concentrations also capture the acute response to surgical injury. Recent data indicate that CRP measured 48 h after surgery has moderate predictive value for postoperative atrial fibrillation (AUC = 0.68), which improves when combined with the neutrophil-to-lymphocyte ratio (176). In patients undergoing coronary artery bypass grafting, those with CRP >175 mg/L on postoperative day 4 had a 1.6-fold higher risk of postoperative atrial fibrillation compared with those whose CRP was <90 mg/L (OR = 1.60, 95% CI 1.23-2.08) (177).

CRP is also associated with recurrence after catheter ablation. Numerous studies have identified baseline CRP or hs-CRP as a prognostic marker of AF recurrence after ablation (178–181). A meta-analysis of 17 studies showed that elevated pre-ablation hs-CRP was associated with increased recurrence risk (OR = 1.86, 95% CI 1.45-2.39) (180). In patients with psoriasis and AF, both preoperative CRP (aHR = 1.2, P = 0.016) and psoriasis history (aHR = 2.3, P = 0.046) emerged as independent predictors of atrial tachycardia recurrence (178). Dynamic changes in CRP after ablation also carry prognostic value. In a small prospective study, CRP remained elevated for several weeks after AF ablation, and an increase from baseline was more frequent among patients with early AF recurrence (181).

Composite laboratory indices have also been investigated for predicting recurrence after catheter ablation. The CRP-to-albumin ratio, uric acid-to-albumin ratio, and systemic immune-inflammation index have all been identified as independent predictors of recurrence after cryoablation, with the CRP-to-albumin ratio showing particularly strong performance (182). In hypertensive patients, the systemic immune-inflammation index outperformed CRP and the neutrophil-to-lymphocyte ratio in predicting post-ablation recurrence (AUC = 0.72 vs. 0.65 vs. 0.63) (183). These composite indices may complement established clinical and echocardiographic variables, although their incremental value requires validation in larger cohorts.

### 5.2 CRP and other types of arrhythmias

Although most research has focused on atrial fibrillation, circulating CRP has also been examined in ventricular arrhythmias and conduction system disease. The evidence in these settings is less consistent and is derived mainly from observational studies.

#### 5.2.1 Ventricular arrhythmias and sudden cardiac death

Ventricular arrhythmias (VAs), including ventricular tachycardia (VT) and ventricular fibrillation (VF), are major causes of sudden cardiac death (SCD).

The relationship between circulating CRP and ventricular arrhythmias remains uncertain. In a prospective study of 489 patients with an implantable cardioverter-defibrillator, CRP was associated with heart failure hospitalization and mortality but not with incident device-detected ventricular arrhythmias; cardiac troponin T, rather than CRP or IL-6, independently predicted ventricular arrhythmia risk (184).

Associations between CRP and ventricular repolarization have been reported in acute inflammatory conditions. In patients with COVID-19, higher circulating CRP correlated with QTc prolongation and other indices of abnormal ventricular repolarization (185, 186). These findings indicate that CRP may reflect the systemic inflammatory and myocardial stress accompanying repolarization abnormalities, but its value as a ventricular arrhythmia-specific biomarker remains less established than its role in AF.

#### 5.2.2 Conduction system diseases

Conduction system diseases, including atrioventricular block and bundle branch block, can cause bradycardia and cardiac syncope, with severe cases requiring pacemaker implantation. In a prospective cohort of 4,314 older adults, Frimodt-Møller et al. found that higher baseline hs-CRP was associated with incident conduction disease over a median follow-up of 7 years. After multivariable adjustment, each 10-mg/L increase in hs-CRP was associated with a 7% higher risk of conduction disease (HR = 1.07, 95% CI 1.00-1.14), and the association was most evident at higher hs-CRP concentrations (187). Inflammation and fibrosis are biologically relevant to conduction system degeneration, but current clinical evidence primarily supports hs-CRP as a circulating marker of inflammatory risk in this setting (187).

### 5.3 Anti-inflammatory and metabolic interventions in cardiac arrhythmias

Several anti-inflammatory, lipid-lowering, metabolic, and lifestyle interventions can reduce circulating CRP while influencing arrhythmia-related outcomes. These treatments modify inflammatory burden indirectly and do not specifically target CRP.

#### 5.3.1 Anti-inflammatory drug therapy

Statins are the most extensively studied lipid-lowering agents with additional anti-inflammatory effects in this setting. A meta-analysis of cohort studies found that atorvastatin therapy lowered hs-CRP in patients with AF, whereas randomized evidence has mainly examined the prevention of postoperative AF (188, 189). Earlier meta-analyses reported a lower incidence of postoperative AF with perioperative statin therapy, although subsequent analyses restricted to trials at low risk of bias did not confirm this benefit (189, 190). The underlying mechanisms may involve lipid lowering, modulation of inflammatory and oxidative pathways, and effects on the postoperative atrial substrate.

Colchicine has been evaluated for the prevention of postoperative AF, with inconsistent results across trials. In the Colchicine for the Prevention of the Post-Pericardiotomy Syndrome (COPPS) AF substudy, colchicine reduced postoperative AF from 22% to 12% (191). In contrast, the COPPS-2 randomized trial found no significant reduction in postoperative AF in the intention-to-treat analysis and reported more adverse events, predominantly gastrointestinal intolerance (192).

#### 5.3.2 Novel glucose-lowering agents

Sodium-glucose cotransporter 2 (SGLT2) inhibitors have demonstrated cardiovascular benefits beyond glucose lowering. In patients with diabetes treated with an implantable cardioverter-defibrillator or cardiac resynchronization therapy defibrillator, SGLT2 inhibitor use was associated with improvements in electrophysiological indices and lower arrhythmia burden, together with changes in CRP and sympathetic activity (193). In patients undergoing coronary artery bypass grafting, a small randomized trial found that perioperative empagliflozin reduced postoperative CRP and ventricular arrhythmias, whereas the reduction in postoperative AF was not statistically significant (194).

Glucagon-like peptide-1 receptor agonists (GLP-1RAs) also exhibit notable anti-inflammatory and cardiovascular protective effects. In the STEP-HFpEF program, semaglutide treatment significantly lowered serum CRP levels in patients with obesity-related heart failure with preserved ejection fraction (HFpEF) while improving symptoms and quality of life (195). The benefit in the Kansas City Cardiomyopathy Questionnaire Clinical Summary Score was more pronounced in patients with concomitant AF than in those without AF (195). This analysis evaluated HF-related health status according to baseline AF status, supporting semaglutide as an indirect modifier of metabolic and inflammatory burden rather than a CRP-directed antiarrhythmic therapy.

#### 5.3.3 Lifestyle interventions

Lifestyle modifications form the foundation for management of modifiable AF risk factors. Weight loss, regular exercise, smoking cessation, limited alcohol intake, and a healthy diet can improve metabolic health and reduce systemic inflammatory burden. In the Long-Term Effect of Goal-Directed Weight Management in an Atrial Fibrillation Cohort (LEGACY) study, sustained weight loss was associated with a dose-dependent improvement in long-term freedom from AF, whereas weight fluctuation attenuated this benefit (196). In patients with obstructive sleep apnea, continuous positive airway pressure therapy has been associated with reduced CRP levels and fewer nocturnal arrhythmic events (173). These interventions act through overlapping metabolic, hemodynamic, autonomic, and inflammatory pathways.

#### 5.3.4 Evidence gaps and future directions

Li et al. analyzed data from 86,424 individuals and found no significant causal association between genetically predicted CRP levels and AF risk (OR = 1.02, 95% CI 0.97-1.07, P = 0.45) (197). This genetic evidence complements the clinical literature by distinguishing associations with circulating CRP from the inflammatory pathways accompanying AF.

Future studies should standardize the timing and assay methods used for CRP and hs-CRP measurements before and after cardiac surgery or ablation. Conformation-specific studies are also needed to determine whether pCRP*, mCRP, or both accumulate in atrial tissue and participate in local remodeling. Randomized anti-inflammatory trials incorporating predefined AF incidence or recurrence endpoints, serial inflammatory measurements, and mediation analyses may further clarify whether changes in CRP track or mediate treatment effects.

In summary, CRP is one of the most extensively studied circulating inflammatory biomarkers in arrhythmia research. Circulating CRP and hs-CRP have the most consistent clinical value in AF, particularly for estimating the risks of incident AF, postoperative AF, post-ablation recurrence, and adverse cardiovascular outcomes. Experimental CRP exposure studies support links with inflammatory and profibrotic signaling in atrial cells, whereas conformation-specific evidence from human atrial tissue remains limited. Current anti-inflammatory and metabolic therapies influence CRP indirectly, and CRP-directed treatment has not been established for arrhythmia management.

## 6 CRP in cardiomyopathies

Cardiomyopathies comprise a heterogeneous group of myocardial disorders in which systemic and myocardial inflammation varies according to etiology, phenotype, and disease stage. Circulating CRP, most often measured using high-sensitivity CRP (hs-CRP) assays, has been evaluated primarily as a marker of inflammatory activity and clinical risk. Experimental studies also suggest that CRP may exert biological effects during myocardial injury and remodeling under specific conditions (198, 199). This section examines clinical and experimental evidence across major cardiomyopathy phenotypes and summarizes inflammation-modulating and investigational CRP-directed interventions.

### 6.1 CRP in HFpEF-related cardiomyopathy: focus on the obesity-inflammation phenotype

Obesity-related HFpEF can be viewed as a systemic metabolic-inflammatory phenotype with prominent myocardial and coronary microvascular involvement. As detailed in Sections 4.1.3 and 4.3.2, visceral adipose-tissue inflammation, impaired nitric oxide signaling, microvascular dysfunction, and diffuse myocardial fibrosis provide the principal pathophysiological context in which hs-CRP is elevated. Within the cardiomyopathy spectrum, the relevance of CRP therefore lies mainly in identifying an inflammatory-fibrotic phenotype rather than defining a separate disease entity (102, 105, 108).

Clinical evidence from the STEP-HFpEF program and the SUMMIT trial is discussed in Section 4.3.2. Taken together, these studies show that incretin-based treatment improves symptoms and functional status while lowering CRP in patients with obesity-related HFpEF. CRP may consequently assist with inflammatory phenotyping and longitudinal assessment, although current evidence does not support its use as a stand-alone diagnostic marker or as a validated companion test for selecting incretin-based therapy.

### 6.2 CRP in inflammatory cardiomyopathy

Inflammatory cardiomyopathy encompasses a group of disorders characterized by myocardial dysfunction due to myocardial inflammation, including acute myocarditis, chronic inflammatory cardiomyopathy, and autoimmune myocarditis. Circulating CRP and hs-CRP have been investigated as adjunct markers of systemic inflammatory activity, disease course, and prognosis in these conditions (200).

#### 6.2.1 Acute myocarditis and fulminant myocarditis

Acute myocarditis is an important cause of sudden death and heart failure in young adults. Because its clinical presentation is highly variable, evaluation integrates clinical findings, cardiac troponin, electrocardiography, cardiac imaging, and, in selected patients, endomyocardial biopsy. Serum CRP or hs-CRP is frequently elevated and may reflect the systemic inflammatory burden, although its concentration varies with etiology, disease stage, and extracardiac inflammation (200). Fulminant myocarditis (FM), the most severe clinical presentation, is characterized by rapid hemodynamic deterioration, acute heart failure, and cardiogenic shock (201).

Etiologic classification requires microbiological, immunological, imaging, and, when appropriate, histological evaluation. Current evidence does not support fixed CRP thresholds for distinguishing infectious from immune-mediated or drug-induced myocarditis. A meta-analysis comparing FM with non-fulminant myocarditis found no significant between-group difference in CRP levels (202).

Serial hs-CRP measurements may complement clinical findings, cardiac injury biomarkers, and imaging during follow-up, although their role in directing immunomodulatory treatment has not been established (200). Cardiomyopathy-specific intervention studies are discussed in Section 6.6.

#### 6.2.2 Immune checkpoint inhibitor-associated and SARS-CoV-2-related myocarditis

Immune checkpoint inhibitor-associated myocarditis (ICI-M) has emerged as one of the most serious adverse effects of cancer immunotherapy, with its incidence increasing as the use of immune checkpoint inhibitors expands. The clinical presentation of ICI-M varies widely, ranging from asymptomatic biomarker elevation to fatal fulminant myocarditis. Serum hs-CRP may provide complementary information on systemic inflammatory activity, whereas diagnostic assessment relies on cardiac troponin, electrocardiography, cardiac imaging, and the clinical context (203). A recent study identified serum Rho-associated coiled-coil-containing protein kinase 2 (ROCK2) as a candidate diagnostic marker and also observed elevated hs-CRP and inflammatory cytokines in patients with ICI-M (204).

Myocarditis associated with SARS-CoV-2 infection and myocarditis occurring after COVID-19 vaccination represent distinct clinical settings. In reported infection-associated cases, CRP levels vary with the systemic inflammatory response and are interpreted together with cardiac injury biomarkers and cardiac magnetic resonance findings (205). Post-vaccination myocarditis has been reported predominantly in adolescent and young adult men following mRNA vaccination; CRP may be elevated but is not a defining diagnostic feature (206, 207).

### 6.3 CRP in dilated cardiomyopathy

Dilated cardiomyopathy (DCM) is characterized by left or biventricular dilation and systolic dysfunction. It is a major cause of heart failure and heart transplantation. Chronic systemic and myocardial inflammation is associated with the development and progression of DCM. Circulating CRP and hs-CRP have been evaluated mainly as markers of disease severity and prognosis, whereas evidence for direct biological effects is derived largely from experimental myocardial injury models (208, 209).

#### 6.3.1 Experimental evidence linking CRP to myocardial injury and remodeling

Experimental evidence linking CRP to myocardial injury has been obtained mainly from ischemia–reperfusion models rather than from DCM-specific models. In rats, elevated serum CRP altered myocardial microRNA expression after ischemia–reperfusion injury (210). In rabbit and rat models, administration or deposition of CRP increased infarct size and was accompanied by complement activation, apoptosis, and mitochondrial injury (211, 212). These findings indicate that CRP can amplify tissue injury in ischemically damaged myocardium, whereas its contribution to nonischemic DCM remains less clearly defined.

#### 6.3.2 Diagnostic and prognostic value

In a retrospective cohort of 622 hospitalized patients with DCM followed for a mean of 2.6 ± 1.6 years, all-cause mortality was higher among patients with hs-CRP >3.90 mg/L than among those with hs-CRP <3.90 mg/L (33.6% vs. 12.8%, P <0.001). After multivariable adjustment, hs-CRP >3.90 mg/L remained associated with all-cause mortality (HR = 1.922, 95% CI 1.236-2.988, P = 0.004) (213).

Composite inflammatory indices have also been investigated in DCM. In a real-world analysis, 2,122 patient records were screened and 913 patients with DCM were included in the final analysis. The CRP-to-lymphocyte ratio (CLR) was associated with worsening cardiac function and mortality and was incorporated into a clinical prediction model (214). Another single-center study evaluated the delta neutrophil index (DNI) and CRP as complementary inflammatory markers for diagnostic and prognostic stratification in DCM (215).

### 6.4 CRP in diabetic cardiomyopathy

Diabetic cardiomyopathy is a cardiovascular complication of diabetes characterized by myocardial structural and functional abnormalities in the absence of coronary artery disease, hypertension, or other known cardiac conditions. Chronic low-grade inflammation is an important component of diabetic cardiomyopathy. Circulating CRP and hs-CRP have been evaluated as clinical markers of this inflammatory state, whereas CRP-transgenic animal models provide experimental evidence of a potential contribution to diabetic myocardial remodeling (216).

#### 6.4.1 Molecular mechanisms

Hyperglycemia, insulin resistance, and lipid metabolic abnormalities promote systemic inflammation and increase hepatic CRP synthesis. In a streptozotocin-induced diabetes model, human CRP-transgenic mice developed greater left ventricular dysfunction, cardiomyocyte apoptosis, oxidative stress, and myocardial fibrosis than diabetic wild-type mice. The transgenic animals also showed increased expression of inflammatory mediators, renin–angiotensin system components, NADPH oxidase subunits, and connective tissue growth factor (216). These findings provide experimental evidence that sustained human CRP overexpression can aggravate diabetic myocardial remodeling through inflammatory, renin-angiotensin, and oxidative-stress pathways.

#### 6.4.2 Diagnostic and prognostic value

Serum hs-CRP has been examined as an adjunct marker of subclinical myocardial dysfunction in diabetes. In patients with type 2 diabetes without overt heart failure, hs-CRP was associated with subsequent left ventricular diastolic dysfunction, although its predictive performance was weaker than that of soluble suppression of tumorigenicity 2 (217).

In a nested case–cohort analysis of 3,098 participants with type 2 diabetes from the Action in Diabetes and Vascular Disease: Preterax and Diamicron Modified Release Controlled Evaluation (ADVANCE) trial, higher hs-CRP was associated with incident or progressive heart failure after adjustment for established risk factors. However, among hs-CRP, interleukin-6, high-sensitivity cardiac troponin T, and N-terminal pro-B-type natriuretic peptide, only N-terminal pro-B-type natriuretic peptide consistently improved heart failure prediction beyond clinical variables (218).

### 6.5 CRP in other types of cardiomyopathies

#### 6.5.1 Stress cardiomyopathy (Takotsubo syndrome)

Takotsubo syndrome (TTS) is an acute heart failure syndrome triggered by intense emotional or physical stress, characterized by transient apical ballooning of the left ventricle. Inflammatory activation is frequently observed during the acute phase of TTS. In a retrospective cohort, higher serum CRP was associated with lower left ventricular ejection fraction (LVEF) and longer hospital stay, but not with in-hospital complications (219).

Further research has demonstrated the value of CRP in prognostic assessment of TTS. Faucher et al. showed that a CRP level >33 mg/L at discharge was independently associated with 1-year all-cause mortality in patients with TTS. Incorporating discharge CRP into the InterTAK prognostic score improved risk stratification, particularly in intermediate- and very-high-risk patients (220).

#### 6.5.2 Hypertrophic cardiomyopathy

Hypertrophic cardiomyopathy (HCM) is the most common genetic cardiomyopathy, characterized by myocardial hypertrophy, cardiomyocyte disarray, and interstitial fibrosis. Although the primary cause of HCM is mutations in sarcomere protein genes, accumulating evidence indicates that inflammatory responses contribute to disease progression.

In a cross-sectional study, patients with HCM and atrial fibrillation had higher hs-CRP concentrations than patients with HCM without atrial fibrillation (221). In a separate observational cohort of 490 patients with HCM followed for 3.7 ± 2.0 years, elevated plasma hs-CRP was independently associated with cardiovascular death, all-cause mortality, sudden cardiac death, and heart failure-related death (222).

#### 6.5.3 Cancer therapy-related cardiac dysfunction

Anthracyclines and other anticancer treatments can cause cancer therapy-related cardiac dysfunction (CTRCD) through oxidative, inflammatory, and mitochondrial injury pathways (223). Circulating CRP may increase during cancer treatment as part of the systemic inflammatory response. A meta-analysis of 12 studies found a significant increase in CRP after breast cancer treatment, but changes in CRP were not significantly associated with CTRCD risk. Among the inflammatory biomarkers evaluated, only myeloperoxidase showed a significant association with CTRCD (224).

### 6.6 Inflammation-modulating and CRP-directed interventions in cardiomyopathies

Therapeutic approaches can be divided into upstream or indirect inflammation-modulating interventions and investigational strategies that directly remove or target CRP. In current cardiomyopathy studies, reductions in circulating CRP generally reflect suppression of upstream inflammation rather than direct pharmacological inhibition of CRP.

#### 6.6.1 Upstream or indirect inflammation-modulating interventions

The multicenter, open-label, evaluator-blinded phase II HYPIC trial randomized 50 patients with biopsy-confirmed, virus-negative chronic inflammatory cardiomyopathy after FM to hydroxychloroquine plus prednisolone or prednisolone alone for 12 months. The primary composite cardiovascular outcome occurred in 24.0% and 60.0% of patients, respectively (HR = 0.28, 95% CI 0.11-0.71). Combination treatment also produced greater improvements in LVEF, left ventricular internal diastolic diameter, hs-CRP, high-sensitivity cardiac troponin I, and N-terminal pro-B-type natriuretic peptide, with no serious drug-related adverse events reported (225).

In a retrospective series of six patients with chronic active idiopathic myocarditis refractory to standard therapy, add-on anakinra was associated with lower CRP, improved LVEF and functional status, and reduced arrhythmic burden after 3 months. These findings provide preliminary cardiomyopathy-specific evidence for IL-1 inhibition (226).

Colchicine has also been evaluated as adjunctive therapy in myopericarditis. In a retrospective study comparing 33 patients treated with colchicine plus aspirin or a non-steroidal anti-inflammatory drug with 31 patients receiving conventional therapy, changes in CRP and cardiac biomarkers did not differ significantly between groups, whereas improvements in electrocardiographic indices of atrial activation were observed in the colchicine group (227).

Metabolic therapies for obesity-related HFpEF, including semaglutide and tirzepatide, are reviewed in Section 4.3.2. Their effects on CRP are considered indirect and are not repeated here.

#### 6.6.2 Investigational direct CRP removal or targeting

Selective CRP apheresis uses extracorporeal adsorption to remove circulating CRP and can produce a rapid reduction in plasma CRP concentrations (228). Clinical investigation has focused mainly on acute myocardial infarction, whereas its efficacy in myocarditis, DCM, and other cardiomyopathies has not been established in adequately controlled trials.

Pharmacological strategies designed to inhibit CRP binding, conformational conversion, or downstream tissue effects remain investigational. No directly CRP-targeted treatment has established clinical efficacy in cardiomyopathy, and cardiomyopathy-specific evidence for CRP antibodies or small-molecule inhibitors remains preclinical (24).

### 6.7 Evidence gaps and future directions

Methodological issues related to CRP conformation-specific detection and the broader causality debate are discussed in Sections 2.4 and 2.5. In cardiomyopathies, future studies should combine serial hs-CRP measurements with detailed phenotyping, cardiac imaging, myocardial injury biomarkers, and, where available, tissue characterization. Direct assessment of pCRP*, mCRP, and their myocardial distribution will be required to determine whether local conformational conversion contributes to particular cardiomyopathy phenotypes.

Prospective studies should evaluate whether hs-CRP provides clinically meaningful information beyond established diagnostic and prognostic markers and whether treatment-related changes in hs-CRP predict subsequent clinical benefit. Intervention trials should also distinguish upstream suppression of inflammation from direct CRP removal or inhibition. Current evidence supports circulating CRP and hs-CRP as adjunct markers of inflammatory activity and risk in selected cardiomyopathies, whereas experimental studies identify context-dependent biological effects in injured myocardium. Direct CRP-targeted treatment in cardiomyopathy remains investigational.

## 7 CRP in hypertension

Hypertension is the leading modifiable risk factor for cardiovascular mortality worldwide. Its pathogenesis extends beyond hemodynamic abnormalities; chronic low-grade inflammation has been recognized as an important contributor to the initiation, progression, and target-organ damage associated with hypertension (229, 230). Circulating CRP, commonly quantified using hs-CRP assays, is a marker of low-grade systemic inflammation. Observational studies have associated elevated CRP with incident hypertension, hypertension-mediated organ damage, and adverse cardiovascular outcomes, whereas experimental and genetic studies have yielded less consistent evidence regarding causality (231, 232). These associations have also been examined in hypertension accompanied by metabolic abnormalities, gestational hypertension, and resistant hypertension. This section reviews the molecular mechanisms, clinical associations, therapeutic implications, and future directions regarding the role of CRP in hypertension.

### 7.1 Hypertension-specific mechanisms and genetic associations

Vascular endothelial dysfunction is a key initiating event in hypertension. The general effects attributed to CRP conformational forms on endothelial signaling, nitric oxide bioavailability, oxidative stress, and vascular smooth muscle cells are described in Section 2.3. In hypertension, interactions between inflammatory signaling and the renin–angiotensin system (RAS) may be particularly relevant. In transgenic mice expressing rabbit CRP, systolic hypertension was accompanied by reduced vascular angiotensin II type 2 receptor (AT2R) expression and an exaggerated blood pressure response to angiotensin II, whereas vascular angiotensin II type 1 receptor abundance remained unchanged (233).

Increased vascular stiffness and vascular remodeling are central features of hypertension progression. Higher circulating CRP has been associated with arterial stiffness and vascular remodeling in clinical and experimental studies (234). In spontaneously hypertensive rats expressing human CRP, increased aortic oxidative stress was observed without parallel increases in several inflammatory markers, indicating that the vascular effects associated with CRP may depend on the experimental and pathophysiological context (235).

Single-nucleotide polymorphisms (SNPs) in the CRP gene influence baseline circulating CRP concentrations and have been investigated in relation to hypertension susceptibility (236, 237). In a prospective nested case–control study of a Chinese Han population, specific CRP gene polymorphisms were associated with both circulating CRP levels and the risk of essential hypertension, although the magnitude and direction of these associations varied among individual variants (236, 238). A cohort study in a Turkish population further revealed sex-specific effects of CRP haplotypes: the CGCA haplotype in women and the TGTG haplotype in men were associated with higher serum CRP levels and increased hypertension risk, highlighting population- and sex-related heterogeneity in CRP genetic regulation (237). These findings suggest that the relationship between CRP-related genetic variation and hypertension susceptibility is population- and sex-dependent.

### 7.2 Clinical associations of CRP with different hypertension subtypes

Patients with essential hypertension commonly exhibit low-grade inflammatory activation, and higher circulating hs-CRP has been associated with prevalent and incident hypertension (239–242). A case–control study showed that patients with hypertension have higher hs-CRP levels than healthy individuals, with levels in grade 2 hypertension being higher than in grade 1 hypertension, suggesting that inflammatory activity increases with disease severity (243). Large cohort studies have identified elevated baseline hs-CRP as a predictor of incident hypertension in middle-aged and older adults. This association persisted after multivariable adjustment in several cohorts, although adiposity and metabolic dysfunction remained important determinants of both CRP concentration and hypertension risk (240, 241). A recent integrated analysis of three cohorts reported that mediation models attributed part of the associations of the triglyceride-glucose (TyG) index and obesity with hypertension and adverse cardiovascular events to CRP. The estimated mediating effect was greater than that observed for several other inflammatory indices, including the systemic inflammation response index (SIRI) (242).

Circulating CRP levels vary across specific hypertension phenotypes. In pregnancy-related hypertension, women with gestational hypertension have significantly higher hs-CRP levels than those with normal pregnancies (P = 0.043), indicating greater systemic inflammatory activity in this population (244). In H-type hypertension (hypertension with hyperhomocysteinemia), the coexistence of elevated homocysteine and CRP has been associated with a higher risk of recurrent ischemic stroke (245). Patients with resistant hypertension also exhibit higher CRP levels than those with uncomplicated hypertension, and elevated CRP has been associated with higher cardiovascular risk in this population (246). In addition, patients with non-dipper hypertension may exhibit elevated CRP levels and inflammatory activation, suggesting an association between systemic inflammation and abnormal blood pressure circadian rhythms (247, 248).

### 7.3 Clinical value of CRP for risk stratification and hypertension-mediated organ damage

Circulating hs-CRP has been investigated as an adjunct to conventional cardiovascular risk assessment but is not a diagnostic test for hypertension (249). Current European Society of Cardiology guidance bases the diagnosis of hypertension on repeated office or out-of-office blood pressure measurements and recommends established measures of hypertension-mediated organ damage (HMOD) for risk assessment; hs-CRP is not included among routine diagnostic tests (250). Among patients with hypertension and metabolic syndrome, baseline hs-CRP levels have been associated with the likelihood of blood pressure remission, with lower inflammatory status associated with higher remission rates. This association may help characterize the clinical course of metabolically related hypertension (251).

Regarding target-organ damage, elevated hs-CRP has been associated with cardiac, renal, and vascular HMOD (252, 253). Community-based studies in older hypertensive populations have shown that hs-CRP levels are associated with left ventricular hypertrophy (LVH), with 1.25 mg/L identified as a potential threshold for predicting LVH in one study population (252, 254, 255). Prospective studies with 3-year follow-up have reported that elevated hs-CRP independently predicts the onset and progression of microalbuminuria in patients with essential hypertension, supporting an association with hypertensive kidney injury (253, 256). In addition, hs-CRP levels correlate positively with carotid intima-media thickness and the extent of atherosclerotic lesions in hypertensive patients and may complement established measures of vascular target-organ damage (257).

In long-term prognostic assessment, elevated CRP is associated with adverse cardiovascular outcomes in patients with hypertension (258, 259). Data from the China Health and Retirement Longitudinal Study (CHARLS) national cohort showed that individuals with both hypertension and elevated CRP had a 2.7-fold higher risk of incident stroke compared with those who had normal blood pressure and normal CRP levels, indicating a joint association of hypertension and systemic inflammation with stroke risk (258). Recent prospective cohort studies have further demonstrated that even in patients whose blood pressure is well controlled, persistently elevated CRP is associated with increased residual risk of major adverse cardiovascular events (MACE). These findings support the evaluation of residual inflammatory risk alongside blood pressure control (259).

### 7.4 Treatment-associated changes in CRP in hypertension

Several antihypertensive drug classes have been associated with changes in circulating inflammatory markers, potentially through modulation of the RAS, sympathetic activity, endothelial function, and the underlying disease state (260, 261). Small clinical studies of perindopril, bisoprolol, and valsartan have reported reductions in circulating CRP or hs-CRP alongside improvements in blood pressure or microalbuminuria (262, 263). These agents act as upstream or indirect modulators of inflammation rather than as CRP-directed therapies.

Lifestyle and adjunctive interventions that improve metabolic or oral health may influence blood pressure and inflammatory markers concurrently. Dietary lipid modification, periodontal treatment, and exercise-based interventions have been associated with changes in blood pressure and CRP in selected studies (264–266). Their effects on CRP reflect broader metabolic and anti-inflammatory changes rather than direct CRP inhibition.

### 7.5 Causality, evidence gaps, and future directions

Several important controversies remain in the current understanding of the relationship between CRP and hypertension. One major point of debate concerns causality. While some observational studies have reported a significant association between elevated CRP and incident hypertension, other studies have found that this association becomes non-significant after adjustment for confounding factors such as body mass index and insulin resistance. This suggests that the observed relationship may be mediated by shared metabolic and lifestyle factors (266). An earlier Mendelian randomization analysis using CRP-associated genetic variants did not support a causal effect of circulating CRP on blood pressure or hypertension (267). A later two-sample bidirectional Mendelian randomization study reported an association between genetically predicted CRP and hypertensive heart disease (8). Because hypertensive heart disease represents cardiac involvement in the setting of hypertension rather than incident essential hypertension or a quantitative blood pressure trait, additional genetic studies are required to define the direction and scope of this relationship.

A second area of debate relates to the specificity of CRP. As a non-specific inflammatory marker, CRP levels can rise in various conditions, including infection and metabolic disorders. Therefore, CRP alone may not precisely reflect inflammation specifically related to hypertension (231). Serial hs-CRP measurements considered together with ambulatory blood pressure, metabolic variables, renal function, and established measures of HMOD may provide more informative phenotyping than a single measurement.

Future studies should evaluate repeated hs-CRP measurements in ethnically and clinically diverse cohorts, with standardized assessment of ambulatory blood pressure and HMOD. Genetic and metabolic analyses should examine how sex, ancestry, obesity, the TyG index, and insulin resistance modify the association between CRP and hypertension (237, 238, 242, 268). Mechanistic studies should distinguish circulating CRP concentrations measured by conventional CRP or hs-CRP assays from tissue-associated pCRP* and mCRP and determine their relevance to hypertensive vascular and cardiac injury. Intervention studies should establish whether changes in CRP provide prognostic information beyond achieved blood pressure and whether upstream modulation of inflammation improves cardiovascular outcomes.

In summary, elevated circulating CRP or hs-CRP is associated with incident hypertension, selected hypertension phenotypes, HMOD, and adverse cardiovascular outcomes. Experimental studies support context-dependent interactions with vascular and RAS signaling, whereas genetic evidence for a causal effect on hypertension remains inconsistent. Measurement of hs-CRP may therefore contribute to risk refinement in selected populations, but it does not replace established blood pressure measurements or HMOD assessment. Changes in CRP during antihypertensive or lifestyle interventions should be interpreted as indirect effects, and direct CRP-targeted treatment has not been established for hypertension.

## 8 CRP in aortic aneurysm

Aortic aneurysm (AA) is a life-threatening vascular disease characterized by degenerative remodeling of the aortic wall, elastic fiber fragmentation, and abnormal luminal dilation. It can be classified into subtypes such as abdominal aortic aneurysm (AAA), ascending aortic aneurysm (AsAA), and thoracic aortic aneurysm (TAA) according to anatomical location. Aortic aneurysms are frequently asymptomatic before progressive dilation or rupture and are associated with substantial cardiovascular morbidity and mortality (269). Inflammatory cell infiltration, extracellular matrix degradation, vascular smooth muscle cell loss or phenotypic alteration, and impaired repair contribute to progressive weakening of the aortic wall (270, 271). Circulating CRP, commonly measured using high-sensitivity CRP (hs-CRP) assays, has been associated with the presence and selected clinical features of aortic aneurysm. In parallel, conformation-sensitive tissue studies have detected monomeric CRP (mCRP) immunoreactivity in human AAA specimens and have related its distribution to local immune and matrix alterations (270, 272, 273). These clinical, tissue-based, and experimental findings represent distinct levels of evidence. This section reviews the local tissue findings, experimental mechanisms, clinical associations, and therapeutic implications of CRP in aortic aneurysm with particular attention to causality and analytical limitations.

### 8.1 Local CRP deposition and experimental evidence in aortic aneurysm

Conformation-sensitive immunohistochemical studies have identified mCRP immunoreactivity in human AAA specimens, particularly in regions with elastic-layer degeneration (273). The transition from circulating pCRP to tissue-associated pCRP* and mCRP is described in Sections 2.1 and 2.3. In AAA tissue, the origin and accumulation of mCRP may involve deposition and local conformational alteration of circulating CRP, although the relative contribution of locally expressed CRP remains uncertain.

Spatial whole-transcriptomic analysis of human AAA specimens found that regions with greater mCRP immunoreactivity exhibited distinct expression patterns involving acute-phase responses, complement and coagulation pathways, extracellular matrix regulation, and immune-cell signaling (270). These tissue-level associations link mCRP deposition with the inflammatory and structural heterogeneity of the aneurysmal wall.

Multiplexed tissue imaging has further shown that AAA specimens with greater mCRP immunoreactivity contain higher proportions of M1-like and proliferating macrophages, fewer α-smooth muscle actin-positive stromal cells, and distinct spatial relationships among lymphoid, endothelial, and stromal populations (274). Lower-mCRP specimens showed comparatively greater fibrosis and enrichment of selected CD163-positive macrophage populations. Together with the spatial transcriptomic findings, these observations associate tissue mCRP with immune-stromal remodeling rather than with a single uniform macrophage response (270, 274).

Experimental evidence for a functional contribution of CRP has been obtained in a porcine pancreatic elastase-induced mouse model of AAA. Compared with wild-type mice, CRP-deficient mice developed less aortic dilation, retained more medial elastin, and showed reduced macrophage accumulation and matrix metalloproteinase-2 expression (271). CRP deficiency did not produce corresponding changes in smooth muscle cell loss, lymphocyte accumulation, angiogenesis, or matrix metalloproteinase-9 expression, indicating selective effects within this experimental model.

CRP gene variants have also been examined in relation to AAA susceptibility. The rs3091244 polymorphism was associated with AAA in one clinical genetic study (275), whereas another study found that the elevation of circulating CRP in patients with AAA was independent of the principal CRP polymorphism examined (276). Genetic associations therefore vary according to the variant and study population and are considered together with Mendelian randomization evidence in Section 8.5.

### 8.2 Clinical associations of CRP with different aortic aneurysm subtypes

Abdominal aortic aneurysm (AAA) is the most extensively studied subtype of aortic aneurysm in relation to CRP. Case–control studies and meta-analyses have generally reported higher circulating CRP or hs-CRP concentrations in patients with AAA than in controls, with associations involving aneurysm presence, diameter, and inflammatory activity (272, 277). In tissue studies, stronger mCRP immunoreactivity was associated with higher serum CRP and larger maximum aneurysm diameter (273). Evidence relating circulating CRP to subsequent expansion is less consistent; one prospective study of small-diameter AAA found that plasma CRP did not reflect aneurysm expansion (278). Follow-up studies of patients after AAA repair have shown that elevated preoperative CRP and persistently high postoperative CRP were associated with aneurysmal disease progression in selected cohorts (279). In patients with infected abdominal aortic aneurysm, CRP levels rise rapidly in response to local infection and may assist in assessing the systemic inflammatory response and monitoring treatment when interpreted with microbiological and imaging findings (280, 281).

Circulating inflammatory markers have also been investigated in AsAA and TAA. In a cross-sectional study, patients with AsAA had higher hs-CRP concentrations and monocyte-to-high-density lipoprotein ratios than controls, supporting an association between systemic inflammation and this phenotype (282). In a small cohort of patients with uncomplicated TAA, the CRP-to-interleukin-6 (CRP/IL-6) ratio was associated with aneurysm size (283). In addition, patients with inflammation-related or autoimmune-associated aortic aneurysm may show marked CRP elevation; this pattern has been reported in IgG4-related vascular disease and reflects the systemic inflammatory component of these conditions (284).

Relationships between circulating CRP and aneurysm activity vary among clinical settings and cannot be represented by a single pattern of stable or progressive disease (272, 278). Aortic aneurysm intraluminal thrombus burden also correlates with CRP levels; thicker thrombi are associated with higher serum CRP, MMP-9, and homocysteine concentrations (285). In addition, post-implantation syndrome following endovascular aortic repair is characterized by a sharp postoperative rise in CRP, fever, and systemic inflammatory activation. Peak CRP levels are associated with the intensity of the postoperative systemic inflammatory response and have been investigated in relation to the risk of short-term complications (286).

### 8.3 Clinical and prognostic associations of circulating CRP

Ultrasonography, computed tomography, and magnetic resonance imaging remain the principal methods for detecting and monitoring aortic aneurysms (269). Circulating hs-CRP is neither specific to aortic inflammation nor established as a standalone screening or diagnostic test. Higher hs-CRP concentrations have nevertheless been associated with AAA in observational studies, and combined measurement of hs-CRP and cathepsin S has been explored as an indicator of inflammatory activity within AAA tissue (277, 287). No universally validated hs-CRP threshold is available for aneurysm detection. In suspected infected aneurysm, marked CRP elevation may support assessment of systemic inflammation but must be interpreted together with clinical, microbiological, and imaging findings (280, 281).

For risk assessment, circulating CRP has been related to aneurysm diameter, tissue mCRP immunoreactivity, and selected measures of vascular stiffness (272, 273, 288). The CRP/IL-6 ratio has also been associated with TAA size (283). These measures remain investigational and have not replaced established determinants such as aneurysm diameter, expansion rate, symptoms, anatomical morphology, and underlying genetic aortopathy.

The prognostic evidence is heterogeneous. Elevated CRP was associated with aneurysmal disease progression after endovascular repair in one cohort (279), whereas plasma CRP did not reflect expansion of small-diameter AAA in another study (278). Circulating biomarkers, including CRP, were also not associated with endoleak after endovascular repair in a separate cohort (289). Serial CRP measurements may assist in evaluating postoperative inflammation or infection, but their prognostic value depends on the procedure, timing of measurement, and clinical outcome considered (279, 286, 289).

### 8.4 Inflammation-modulating and investigational interventions in aortic aneurysm

Current medical management emphasizes control of cardiovascular risk factors rather than direct CRP inhibition. Statins, blood pressure control, antiplatelet therapy when otherwise indicated, and smoking cessation are used to reduce overall cardiovascular risk in patients with aortic aneurysm. Observational evidence suggests that statins may be associated with slower AAA growth and improved perioperative outcomes, whereas evidence for specific effects of renin–angiotensin system inhibitors on aneurysm expansion remains limited or inconsistent (269). Any accompanying reduction in CRP represents an indirect effect of broader vascular and inflammatory modulation.

Sodium-glucose cotransporter 2 inhibitors have shown potentially protective associations in diabetes-related observational and experimental studies, with proposed mechanisms involving oxidative stress and inflammation; their effect on aneurysm progression has not been established in randomized trials (290). The BANBOO randomized trial was designed to evaluate rivaroxaban in patients with AAA and elevated hs-CRP, but the published report is a study protocol and does not provide evidence of efficacy (291).

Doxycycline has been investigated because of its effects on matrix metalloproteinases and acute-phase reactants (292). However, a multicenter randomized clinical trial found no reduction in small infrarenal AAA growth after 2 years of doxycycline treatment compared with placebo (293).

Smoking cessation is a central component of aneurysm risk management because smoking is strongly associated with AAA development and rupture, with particularly marked relative risk in women (294). Exercise, dietary measures, and management of hypertension, diabetes, and dyslipidemia support overall cardiovascular health, but evidence that lowering CRP through these measures independently slows aneurysm expansion is lacking.

After endovascular aneurysm repair (EVAR) or open repair, serial CRP measurements can contribute to the assessment of postoperative inflammatory responses, post-implantation syndrome, and possible infection (286, 295). Management is directed toward the underlying postoperative condition rather than CRP concentration itself. No direct CRP removal or conformation-specific CRP inhibitor has established clinical efficacy in aortic aneurysm.

### 8.5 Causality, limitations, and future directions

The causal relationship between CRP and aortic aneurysm remains unresolved. Observational studies associate higher circulating CRP with AAA presence and selected measures of disease severity, but these relationships may be influenced by smoking, atherosclerosis, adiposity, infection, and preexisting tissue injury (272, 277, 278). A Mendelian randomization analysis found that genetically predicted elevation of circulating CRP was not associated with AAA risk, despite a positive conventional observational association (296). By contrast, CRP deficiency attenuated aneurysm formation in an elastase-induced mouse model (271), and conformation-sensitive studies have identified mCRP immunoreactivity in human AAA tissue (270, 273, 274). These findings support context-dependent experimental and tissue-level involvement while distinguishing it from the epidemiological association of circulating CRP.

The limited specificity of circulating CRP also constrains clinical interpretation. Infection, autoimmune disease, metabolic dysfunction, smoking, atherosclerosis, and recent surgery can all increase CRP independently of aneurysm activity. In addition, AAA, degenerative TAA, heritable thoracic aortic disease, inflammatory aortopathy, and infected aneurysm have distinct biological backgrounds. Available CRP studies remain concentrated in AAA and cannot be generalized uniformly across these phenotypes (272, 282, 284).

Future studies should combine repeated circulating hs-CRP measurements with standardized imaging of aneurysm diameter and growth, clinical outcomes, and conformation-sensitive analysis of resected tissue. Prospective studies across anatomical subtypes, sexes, ancestries, and heritable aortic disorders are needed to determine whether CRP adds prognostic information beyond established clinical and imaging variables. Spatial transcriptomics and single-cell imaging may further define the immune and stromal environments associated with mCRP deposition (270, 274). Evaluation of direct CRP removal or conformation-specific inhibition would be required to determine whether CRP itself represents a therapeutically modifiable component of aneurysm biology.

In summary, circulating CRP or hs-CRP is associated with AAA presence, tissue inflammation, and selected clinical outcomes, but it is not an established screening or diagnostic test for aortic aneurysm. Conformation-sensitive studies have identified mCRP immunoreactivity in human AAA tissue, and CRP deficiency attenuates aneurysm formation in a specific experimental model. Human genetic evidence has not established a causal effect of circulating CRP on AAA. CRP may therefore serve as an adjunct marker of inflammatory activity in selected settings, whereas its value as a direct therapeutic target in aortic aneurysm remains investigational.

## 9 Future perspectives

### 9.1 CRP in cardiovascular disease: unresolved questions and translational priorities

Over nearly a century of investigation, CRP has evolved from a non-specific marker of acute inflammation into a widely used biomarker of systemic inflammatory burden and cardiovascular risk. Experimental studies have further identified conformation-dependent biological effects of pCRP* and mCRP, although their causal and clinical significance remains incompletely defined. This review has examined CRP across six major cardiovascular phenotypes: coronary artery disease, heart failure, arrhythmias, cardiomyopathies, hypertension, and aortic aneurysm. Across these conditions, circulating CRP measured by hs-CRP assays is most consistently associated with risk and prognosis, whereas the strength of tissue-level and experimental evidence varies by disease. Trials such as CANTOS and STEP-HFpEF demonstrate that selected inflammatory and metabolic pathways are therapeutically modifiable but do not establish CRP itself as the molecular target responsible for clinical benefit. Future translation therefore depends on clearer causal inference, standardized conformation-specific measurement, reproducible inflammatory phenotyping, and rigorous evaluation of direct CRP-directed interventions.

### 9.2 Integration of key scientific findings and conceptual shifts

#### 9.2.1 Evolving interpretation of CRP: systemic biomarker and potential local effector

As detailed in Section 2, the principal conceptual advance is the recognition that the biological activity of CRP is both conformation and context dependent. The translational implication is that circulating hs-CRP, local tissue deposition, and conformationally altered CRP should not be treated as interchangeable measures. This distinction provides a more cautious framework for interpreting the strong prognostic value of hs-CRP alongside the still-unresolved evidence for direct CRP-mediated tissue injury.

#### 9.2.2 Disease-specific roles of CRP across cardiovascular phenotypes

Across cardiovascular phenotypes, the most consistent role of hs-CRP is as a marker of systemic inflammatory burden and adverse prognosis, whereas the strength and nature of mechanistic evidence vary by disease. Plaque inflammation predominates in coronary artery disease, comorbidity-driven microvascular and myocardial remodeling in HFpEF, electrical and structural remodeling in atrial fibrillation, and immune-matrix remodeling in aortic aneurysm. These differences favor disease-specific interpretation of CRP over a single universal pathogenic model and provide a more defensible basis for inflammatory phenotype–guided risk assessment.

#### 9.2.3 From single-marker assessment to multidimensional composite systems

The composite indices reviewed in the disease-specific sections illustrate a broader shift from isolated CRP measurement toward integrated assessment of inflammation, metabolism, nutrition, and immune status. Their main potential lies in improving phenotypic resolution when hs-CRP alone lacks specificity; however, external validation, standardized thresholds, and demonstration of incremental value beyond established clinical models remain necessary before routine implementation.

### 9.3 Core controversies and unresolved scientific questions

Although research on CRP in cardiovascular disease has advanced considerably, several longstanding controversies and unresolved scientific questions remain. Addressing these issues will be critical in shaping the future direction and clinical translation of CRP research.

#### 9.3.1 Outstanding questions in causal inference

The evidence informing CRP causality is reviewed in Section 2.5. The principal unresolved question is whether one or more CRP conformations contribute to cardiovascular injury independently of the inflammatory, metabolic, and tissue-injury pathways that increase CRP production. Genetic studies predominantly evaluate determinants of circulating CRP, whereas tissue studies describe local pCRP*, mCRP, or CRP immunoreactivity. A direct quantitative relationship between these circulating and tissue-associated pools has not been established.

Future causal research should combine repeated circulating measurements with conformation-specific tissue analysis, longitudinal imaging, appropriately designed experimental models, and clinical outcomes. Interventions that directly remove CRP, prevent conformational transition, or block a defined CRP interaction will be particularly informative because reductions in CRP during upstream anti-inflammatory or metabolic treatment cannot isolate a CRP-specific effect.

#### 9.3.2 Standardization and *in vivo* mapping of CRP conformational forms

Routine CRP and hs-CRP assays do not distinguish pCRP, pCRP*, and mCRP, and current research methods are summarized in Section 2.4. Translation of conformation-specific measurement will require validated antibody specificity, standardized reference materials and calibrators, harmonized preanalytical procedures, and reproducibility across laboratories. Particular attention is needed to prevent sample processing, immobilization, storage, or tissue fixation from altering CRP conformation or epitope accessibility.

Non-invasive assessment of tissue-associated CRP will require molecular probes with well-defined conformational selectivity, suitable pharmacokinetics, and low off-target binding. Imaging signals should be validated against conformation-specific tissue staining and related prospectively to disease activity and clinical outcomes. These steps are necessary before pCRP*, mCRP, or local CRP deposition can be incorporated into cardiovascular classification or treatment selection.

#### 9.3.3 Population heterogeneity: challenges for universal thresholds and personalized approaches

Circulating CRP concentrations vary according to ancestry, sex, age, adiposity, smoking, metabolic status, medication use, genetic background, and comorbid disease. Fixed hs-CRP thresholds may therefore differ in calibration across populations and clinical settings. CRP-associated genetic variants can influence baseline concentrations, but their effects do not necessarily translate into proportional differences in cardiovascular risk or treatment response.

Future studies should use large multi-ancestry cohorts, repeated hs-CRP measurements, and standardized outcome definitions to evaluate population-specific calibration and discrimination. Any proposed threshold or composite model should demonstrate incremental value beyond established clinical predictors and undergo external validation before implementation.

### 9.4 Current status and bottlenecks in clinical translation

#### 9.4.1 Clinical use of hs-CRP: established roles and limitations

Measurement of hs-CRP may refine cardiovascular risk assessment in selected primary- and secondary-prevention settings, but it is not a disease-specific diagnostic test. Interpretation is limited by biological variability and by elevations related to acute infection, autoimmune disease, obesity, smoking, metabolic dysfunction, renal disease, recent procedures, and other inflammatory conditions. Routine assays also provide no information about CRP conformation or tissue source.

Clinical use requires a predefined pathway linking measurement to management. This includes excluding transient inflammatory conditions, repeating measurements when appropriate, and integrating hs-CRP with lipid levels, blood pressure, glycemic status, renal function, established cardiovascular disease, and conventional risk models. More specific tissue or conformational biomarkers remain investigational.

#### 9.4.2 Upstream inflammation modulation and investigational direct CRP targeting

Therapies associated with lower circulating CRP can be divided into upstream or indirect interventions and direct CRP-directed approaches. Upstream interventions include canakinumab, which targets interleukin-1β (16); anakinra, an interleukin-1 receptor antagonist (151–153); tocilizumab, which targets the interleukin-6 receptor; ziltivekimab, which targets the interleukin-6 ligand; and colchicine, which modifies microtubule-dependent and inflammasome-related processes (90–92). In a phase 2 trial involving patients with non-ST-elevation myocardial infarction, tocilizumab attenuated hs-CRP and troponin T release (297). In the phase 2 RESCUE trial, ziltivekimab produced dose-dependent reductions in hs-CRP and other inflammatory and thrombotic biomarkers in patients with chronic kidney disease and elevated hs-CRP (298). Its effects on cardiovascular outcomes are being evaluated in ZEUS (299). Statins (87, 88), glucagon-like peptide-1 receptor agonists (147–149, 195), and sodium-glucose cotransporter 2 inhibitors (145, 146, 193, 194) can also lower CRP indirectly through lipid, metabolic, renal, or systemic anti-inflammatory effects. None of these treatments directly binds or removes CRP, and the strength of cardiovascular outcome evidence differs among agents and clinical settings. CANTOS is discussed in detail in Section 3.5.

Direct CRP-directed approaches include selective CRP apheresis, anti-CRP antibodies, and small molecules designed to alter CRP ligand binding or conformational transition. Selective CRP apheresis rapidly removes circulating CRP but is not conformation-specific. In acute myocardial infarction, the available clinical evidence is based principally on the exploratory, non-randomized CAMI-1 pilot study (300). The randomized CRP-STEMI trial has been designed to evaluate whether selective CRP apheresis reduces infarct size after ST-elevation myocardial infarction (61).

Experimental small molecules such as 1,6-bis(phosphocholine)-hexane can stabilize pCRP and inhibit conformational dissociation in preclinical models; however, their pharmacokinetic suitability and clinical efficacy have not been established in humans (42). Antibody-based approaches remain preclinical. An anti-mCRP monoclonal antibody has shown proof-of-concept anti-inflammatory activity in murine arthritis and nephritis models, although cardiovascular efficacy has not been established (301). Other conformation-selective strategies also remain experimental.

#### 9.4.3 Precision anti-inflammatory therapy: identifying patients likely to benefit

Inflammatory phenotyping may improve the selection and monitoring of patients receiving pathway-specific anti-inflammatory treatment. CANTOS enrolled patients with previous myocardial infarction and baseline hs-CRP concentrations of at least 2 mg/L. In a secondary analysis, participants treated with canakinumab who achieved an on-treatment hs-CRP concentration below 2 mg/L had greater reductions in cardiovascular events than those whose concentrations remained at or above 2 mg/L (16, 302). Thus, baseline hs-CRP defined residual inflammatory risk, whereas the treatment-associated response provided additional prognostic information.

A single hs-CRP measurement is insufficient to characterize the biological diversity of cardiovascular inflammation. More informative phenotyping may combine repeated hs-CRP measurements with clinical context, comorbidities, additional inflammatory markers, and validated imaging or molecular data. Prospective trials should determine whether these approaches improve treatment selection, dosing, duration, safety, and clinical outcomes beyond conventional risk assessment.

### 9.5 Future research priorities

Future CRP research should prioritize questions that connect molecular form, analytical validity, causal inference, and clinically relevant outcomes. Five areas are particularly important.

#### 9.5.1 Systematic elucidation of CRP conformational biology

Mechanistic studies should define the physiological conditions governing the transition from pCRP to pCRP* and mCRP, the persistence and clearance of these forms *in vivo*, and their interactions with membranes, extracellular vesicles, complement, and immune cells. Standardized reagents and conformation-specific assays will be required to compare their distribution and biological effects across cardiovascular phenotypes.

#### 9.5.2 Molecular imaging and non-invasive assessment of local inflammation

Molecular imaging may provide a means of localizing tissue-associated CRP without relying solely on circulating concentrations. Candidate positron emission tomography (PET), single-photon emission computed tomography (SPECT), or multimodal probes will require validation of conformational specificity, tissue penetration, pharmacokinetics, and off-target binding. Their clinical value should be evaluated against histological findings, established imaging measures, and prospective cardiovascular outcomes.

#### 9.5.3 Multi-omics-driven analysis of CRP regulatory networks

Integrated genomic, transcriptomic, proteomic, metabolomic, and epigenomic studies can identify cellular and molecular networks associated with CRP production, conformational alteration, and tissue deposition. Single-cell and spatial approaches may clarify which immune, vascular, and stromal populations coexist with local CRP forms. Genetic and functional analyses should then test whether these relationships are causal or reflect shared inflammatory and metabolic pathways.

#### 9.5.4 Clinical translation of precision anti-inflammatory therapy

Clinical trials should distinguish upstream inflammation-modulating treatment from direct CRP-directed intervention and incorporate evidence of target engagement appropriate to each approach. Direct strategies require demonstration that CRP removal, conformational stabilization, or blockade of a defined CRP interaction produces clinically meaningful benefit. Trials should prespecify inflammatory phenotypes, assess cardiovascular outcomes and safety, and determine whether biomarker-guided selection improves absolute treatment benefit.

#### 9.5.5 Longitudinal inflammatory risk and primary prevention

Longitudinal studies should examine how cumulative inflammatory exposure across the life course relates to cardiovascular risk and whether repeated hs-CRP measurements improve prediction beyond established factors. Diet, physical activity, smoking cessation, weight management, and control of metabolic risk remain the appropriate foundation for prevention. Pharmacological anti-inflammatory treatment and population-wide CRP screening require evidence of net clinical benefit before broader preventive use.

Overall, the future clinical value of CRP will depend on maintaining a clear distinction between systemic inflammatory risk measured by hs-CRP and the potential local effects of specific CRP conformations. Standardized measurement, appropriately designed causal studies, and target-specific intervention trials are required before tissue-associated CRP can be incorporated into clinical classification or direct therapeutic strategies. Until such evidence is available, hs-CRP remains principally a biomarker of inflammatory risk, whereas direct CRP targeting remains investigational.
